# Combination of long-acting TRAIL and tumor cell-targeted photodynamic therapy as a novel strategy to overcome chemotherapeutic multidrug resistance and TRAIL resistance of colorectal cancer

**DOI:** 10.7150/thno.51193

**Published:** 2021-02-25

**Authors:** Tianshan She, Qiuxiao Shi, Zhao Li, Yanru Feng, Hao Yang, Ze Tao, Heng Li, Jie Chen, Shisheng Wang, Yan Liang, Jingqiu Cheng, Xiaofeng Lu

**Affiliations:** 1 Key Lab of Transplant Engineering and Immunology, MOH, Regenerative Medical Research Center, West China Hospital, Sichuan University, Chengdu 610041, China.; 2 Frontiers Science Center for Disease-related Molecular Network, West China Hospital, Sichuan University, Chengdu 610041, China.; 3 West China-Washington Mitochondria and Metabolism Research Center, West China Hospital, Sichuan University, Chengdu 610041, China.; 4 Histopathology Platforms of Experimental Center, West China Hospital, Sichuan University, Chengdu 610041, China.

**Keywords:** Tumor necrosis factor-related apoptosis-inducing ligand, Photodynamic therapy, Multidrug resistance, Cancer targeted therapy, Colorectal cancer.

## Abstract

Chemotherapeutic multidrug resistance (MDR) is the major hindrance for clinical therapy of colorectal cancer (CRC). Tumor necrosis factor-related apoptosis-inducing ligand (TRAIL) with selective cytotoxicity might overcome MDR of CRC cells. Unfortunately, cross-resistance to TRAIL has been detected in many CRC cells, suggesting the need to combine TRAIL with sensitizers to combat refractory CRC. Our purpose is to explore the potential of combination therapy of TRAIL and tumor-cell targeted photodynamic therapy (PDT) in combating CRC with both chemotherapeutic MDR and TRAIL resistance.

**Methods:** Tumor cell-targeted PDT was performed using a Ze-IR700 photosensitizer with high affinity for epidermal growth factor receptor (EGFR). The impact of PDT on the gene expression of CRC cells was revealed by RNA sequencing. The synergistic antitumor effect of long-acting TRAIL and PDT was evaluated in mice bearing tumor grafts of CRC cells with both chemotherapeutic MDR and TRAIL resistance.

**Results:** Chemotherapeutic MDR and TRAIL resistance are common in CRC cells. Pretreatment of CRC cells with tumor cell-targeted PDT significantly (10-60 times) increased the sensitivity of these CRC cells to TRAIL by upregulating death receptors. Combination therapy, but not monotherapy, of long-acting TRAIL and PDT greatly induced apoptosis of CRC cells, thus efficiently eradicated large (~150 mm^3^) CRC tumor xenografts in mice.

**Conclusions:** Tumor cell-targeted PDT extensively sensitizes CRC cells to TRAIL. Combination therapy of long-acting TRAIL and PDT is promising to combat CRC with both chemotherapeutic MDR and TRAIL resistance, which might be developed as a novel strategy for precision therapy of refractory CRC.

## Introduction

Colorectal cancer (CRC) is one of the most common cancers and ranked as the second leading cause of global cancer-associated deaths in 2019 1. Conventional chemotherapy has been the major adjuvant therapeutic strategy for CRC in past decades. Recently, numerous chemicals targeting epidermal growth factor receptor (EGFR)- or vascular endothelial growth factor receptor (VEGFR)-mediated signaling pathways have been identified, providing new alternatives for CRC therapy 2. However, the chemical efflux pump proteins, *i.e.*, the ATP-binding cassette (ABC) transporters, are ubiquitously expressed in enterocytes 3, 4, making it easier for CRC cells to develop chemotherapeutic multidrug resistance (MDR) for both conventional chemotherapeutics and the novel molecular-targeted chemicals 5. Owing to the differences in cell entry and action mechanisms, macromolecular proteins such as anti-EGFR monoclonal antibody can bypass the MDR pathway for small chemical drugs. However, heterogenic mutations in genes such as KRAS, NRAS and BRAF induce CRC resistance to anti-EGFR treatment 6. Despite the rapid progress of therapeutic strategies, the outlook for the CRC incidence rate is bleak. The number of new CRC cases was approximately 1.8 million in 2018, but the global burden of CRC might increase by up to 60% by 2030 1. Evidently, innovative therapeutic strategies are urgently required for precision therapy of CRC with chemotherapeutic MDR.

Apoptosis is an important mechanism for hemeostasis. Tumor necrosis factor-related apoptosis-inducing ligand (TRAIL), a potent apoptosis inducer displayed on the surface of NK cells and cytotoxic T cells, plays important roles in tumor defense 7. Recombinant soluble TRAIL is attractive as an antitumor agent due to its selective apoptosis induction in tumor cells overexpressing TRAIL receptors (death receptor 4, DR4, and death receptor 5, DR5) without systemic toxicity. Although MDR is common in CRC, recombinant TRAIL exerts robust cytotoxicity in numerous CRC cells 8, suggesting the potential of TRAIL for overcoming MDR of CRC. However, the *in vivo* antitumor effect of TRAIL is far from satisfactory, which has been predominantly attributed to the limited tumor uptake resulting from the poor tumor targeting as well as a short serum half-life of TRAIL 9. In past years, a great deal of effort has been made to improve the pharmacokinetics of TRAIL 10-13, which significantly enhanced the antitumor effect of TRAIL to a variety of cancer cells. However, many CRC cells are still resistant to these modified TRAIL formats 8, suggesting the need for combination of TRAIL with a tumor cell sensitizer to overcome chemotherapeutic MDR and TRAIL resistance. In fact, pretreatment with some chemical drugs definitely sensitized CRC cells to TRAIL by upregulating death receptors and/or downregulating anti-apoptotic proteins^14^, ^15^. However, combination of TRAIL and chemotherapeutics did not show a promising synergistic antitumor effect in CRC patients 16. On the one hand, most chemicals combined with TRAIL are nonspecifically toxic to cells. To avoid systemic toxicity, these nonspecific chemicals must be administered at a low dose that might not effectively sensitize tumor cells. On the other hand, the pharmacokinetics of TRAIL and small chemicals are definitely different. As it is time-consuming for chemicals to induce death receptor expression in tumor cells, a short serum half-life would restrict TRAIL to killing tumor cells pre-sensitized by chemicals. Consequently, a combination of long-acting TRAIL and a therapeutic that would selectively act on tumor cells might exert a promising synergistic antitumor effect without systemic toxicity.

Interestingly, it was found that excessive reactive oxygen species (ROS) could sensitize CRC cells to TRAIL by upregulating death receptors and/or downregulating anti-apoptotic proteins 17. Photodynamic therapy (PDT) is efficient in producing ROS by triggering a photosensitizer in cells with laser light 18, 19. In fact, preliminary studies revealed that PDT induced death receptor expression in some cancer cells by producing ROS 20, 21, suggesting that PDT might synergize with TRAIL in killing CRC cells with chemotherapeutic MDR and TRAIL resistance. In addition, due to the unique mechanisms involved, including inducing apoptosis as well as damaging ABC transporters, PDT was considered a potential strategy to overcome MDR of cancers 22, 23. Notably, PDT is achieved by triggering photosensitizer uptake by cells using laser light with a specific wavelength. Due to the short life and limited diffusion distance of ROS, the phototoxicity produced by PDT was confined to cells engulfing photosensitizers and irradiated by laser light. Once the photosensitizers were conjugated to a tumor-homing carrier, the phototoxicity of PDT could be further focused on tumor cells 24, contributing to the precision and biosafety of PDT in cancer therapy. In fact, PDT has been approved for the treatment of various solid tumors, including CRC 25. The potential synergy between PDT and TRAIL in the killing of CRC cells and the clinical biosafety of both PDT and TRAIL greatly triggered our interest in evaluating the effect of their combination in combating CRCs with both chemotherapeutic MDR and TRAIL resistance.

As epidermal growth factor receptor (EGFR) is overexpressed in most CRC cells with MDR, we attempted to perform a tumor-cell targeted PDT by delivering photosensitizers to CRC cells using the Z_EGFR_ affibody, which has high specificity and affinity for EGFR 26, 27. Subsequently, we defined the molecular basis for synergy between tumor-cell targeted PDT and TRAIL in the killing of CRC cells. Finally, we examined the synergistic antitumor effect of tumor cell-targeted PDT and a long-acting TRAIL variant, *i.e.*, IgBD-TRAIL, that could bind endogenous IgG 28 in mice bearing tumor grafts of CRC cells with chemotherapeutic MDR and TRAIL resistance. Our results revealed that the tumor-cell targeted PDT extensively sensitized multidrug-resistant CRC cells to TRAIL by upregulating both DR4 and DR5. Additionally, the combination of the long-acting IgBD-TRAIL, but not TRAIL with short serum half-life, and tumor cell-targeted PDT efficiently eradicated large (~150 mm^3^) tumor grafts of CRC cells with chemotherapeutic MDR and TRAIL resistance, suggesting that combination therapy of long-acting TRAIL and tumor cell-targeted PDT might be a novel strategy for combating refractory CRC.

## Materials and Methods

### Preparation and characterization of Ze affibody

The gene encoding the Z_EGFR_ affibody was designed according to the amino acid sequence of Z_EGFR_:1907 (designated as Ze in this paper) 26. An additional (HE)3 tag and a Cys residue were added at the N- and the C-termini of the Ze affibody, respectively. The gene was optimized for expression in *E. coli* and synthesized by Convenience Biology (Changzhou, China). The expression plasmid pQE30-Ze was constructed by inserting the gene encoding the Ze affibody into pQE30. To produce the Ze affibody, the pQE30-Ze was transformed into *E. coli* M15 and induced by the addition of isopropyl β-D-thiogalactoside (IPTG, 0.1 mM) at 24 °C overnight. After the cells were collected by centrifugation at 7000 g for 10 min, they were resuspended in binding buffer (50 mM phosphate, pH 8.0; 300 mM NaCl; 5 mM imidazole, 1 mM phenylmethylsulfonyl fluoride) followed by passage through a high-pressure homogenizer (50-80 MPa) 3-4 times. The Ze affibodies in the supernatant were bound to Ni-NTA super flow resin (Qiagen, CA) and eluted using elution buffer (50 mM phosphate, pH 8.0; 300 mM NaCl, 300 mM imidazole). The purified Ze affibodies were dialyzed against phosphate-buffered saline (PBS, 10 mM Na_2_HPO_4_, 2 mM KH_2_PO_4_, 137 mM NaCl, 2.68 mM KCl, pH 7.4) overnight, followed by storage at -70 °C until further use.

Sodium dodecyl sulfate polyacrylamide gel electrophoresis (SDS-PAGE) and size exclusion chromatography (SEC) were used to determine the purity and molecular weight of the Ze affibody 29. To measure the affinity of the Ze affibody for EGFR, the extracellular domain of EGFR fused to the Fc of IgG1 (Sino Biological, Beijing, China) was immobilized on protein A-coated probes, followed by insertion of the probe into solutions containing different concentrations of the Ze affibody. The subsequent biolayer interferometry was performed on the Octet® platform (Pall ForteBio LLC, CA) as described by Yang *et al.* 28.

### Preparation and characterization of TRAIL proteins

IgBD-TRAIL and TRAIL were prepared and characterized according to our previous works 28. IgBD-TRAIL was produced by genetically fusing an IgG-binding domain (IgBD) to the N-terminus of TRAIL. To examine the IgG-binding by using SEC, the IgBD-TRAIL was mixed with mouse IgG (mIgG) at a molar ratio of 1:1 followed by incubation at room temperature for 0.5 h prior to loading onto the column for SEC. Formation of a novel protein complex larger than individual mIgG and IgBD-TRAIL in the mixture reflects the binding of IgBD-TRAIL to mIgG. The difference in serum half-life between IgBD-TRAIL and TRAIL was illustrated by measuring the time-dependent reduction in cytotoxicity of the residual TRAIL proteins in the blood. The decreased speed of cytotoxicity reflects the speed of blood clearance of these TRAIL proteins.

### Tumor uptake and tissue distribution of protein

To label the protein, 5(6)-carboxyfluorescein (FAM) or CF^TM^ 750 succinimidyl ester (CF750) (Sigma, MA) was conjugated to the Ze affibody or TRAIL proteins according to the methods described by Shi *et al.* 29. Briefly, the pH of the solution containing 1.5 mg/ml of protein was first adjusted to 8.0 by the addition of 1 M NaHCO_3_. Subsequently, the fluorescent dye dissolved in dimethyl sulfoxide (DMSO) was added to the protein solution at a proper molar ratio (1:20 protein to FAM; 1:6 protein to CF750) followed by incubation at room temperature for 1 h in darkness. The free dye was excluded by dialysis of the mixture against PBS overnight with multiple buffer changes.

To determine the tissue distribution by optical imaging, CF750-labeled proteins (3 mg/kg for Ze and 5 mg/kg for TRAIL proteins) were intravenously injected into mice bearing subcutaneous tumor grafts. Tumors and some important organs/tissues were collected and scanned using an IVIS optical imaging system (Caliper Life Sciences, CA) 4 h later. To further examine the cellular distribution of the Ze affibody, FAM-labeled Ze affibody was intravenously injected into mice. The tumor grafts were collected and sectioned under frozen conditions at 4 h postinjection. Tumor cells were indicated by immunofluorescence with rabbit antibody against human EGFR prior to observation under a fluorescence microscope. Colocalization of FAM-labeled Ze affibody with EGFR reveals the distribution of the Ze affibody on tumor cells.

To investigate the impact of PDT on the tumor uptake of TRAIL proteins, equal molecular amounts of FAM-labeled IgBD-TRAIL or TRAIL were intravenously injected into the mice bearing tumor grafts, immediately followed by PDT. One hour later, the tumor grafts were collected and sectioned under frozen conditions. Cells in tumor tissues were visualized by DAPI staining prior to observation under a multiphoton laser confocal microscope (Nikon, Tokyo, Japan). The uptakes of proteins by tumor grafts treated with or without PDT were compared.

### Conjugation of photosensitizer to the Ze affibody

The photosensitizer IRDye^®^ 700DX N-hydroxysuccinimide ester (IR700, LI-COR Biosciences, Lincoln, NE) was conjugated to the Ze affibody to produce Ze-IR700 according to the method described by Mitsunaga *et al.* 24 with some modifications. Briefly, the Ze affibody was diluted with PBS to 1.5 mg/mL followed by adjustment of the pH of the protein solution to 8.5 by the addition of 1 M Na_2_HPO_3._ Subsequently, the soluble IR700 was mixed with protein at a molar ratio of 1:2 (protein to IR700) and incubated at room temperature for 1 h in darkness. To remove unconjugated IR700 dye, the mixture was dialyzed against PBS overnight with multiple buffer changes. To confirm the conjugation of IR700 to the Ze affibody, the dialyzed mixture was electrophoresed on SDS-PAGE gel and scanned with a LI-COR Odyssey CLx Imager dual-color infrared imaging system (Lincoln, NE).

### Cell culture and cytotoxicity assay

CRC cells, including COLO205, COLO 320 DR, COLO 320 HSR, LS174T, HT29, HCT-8, LOVO, RKO, LS180, T84, and HCT116 cells, were purchased from the American Type Culture Collection (ATCC, VA, USA) and cultured according to the guide supplied by the provider. Short tandem repeat (STR) genotyping of these cells was performed by Feiouer Inc. (Chengdu, China).

To measure the cytotoxicity of TRAIL proteins in CRC cells pretreated with Ze-IR700-mediated PDT, CRC cells (1-2×10^4^ cells/well) seeded in the 96-well clear bottom black plate (Thermo, CA) were incubated with 0.5-2 μM Ze-IR700 at 37 °C for 0.5 h prior to irradiation. After PDT, increasing concentrations of IgBD-TRAIL or TRAIL were added to the cells followed by further incubation overnight. The surviving cells were measured by using CCK-8 solution (Dojindo, Japan) according to the descriptions by Yang *et al.* 28. Compared to the cytotoxicity of TRAIL proteins in CRC cells without PDT treatment, the increase in cytotoxicity of TRAIL in PDT-treated CRC cells reflects the synergy between TRAIL proteins and Ze-IR700-mediated PDT. To determine the death receptor-dependence of the synergy, soluble death receptor including DR4-Fc and DR5-Fc (Sino Biological Inc, Beijing, China) was added into cells treated with PDT prior to addition of TRAIL proteins.

To examine the cytotoxicity of chemical drugs, CRC cells were treated with different concentrations (0-5000 nM) of cisplatin, vincristine, doxorubicin, or bortezomib (Selleck, TX) overnight followed by measuring the surviving cells using CCK8.

To calculate the survival rate, the viability of PBS-treated cells was considered 100%.

### *In vitro* PDT

Ze-IR700-mediated PDT in CRC cells was performed according to the description by Shi *et al.* 29 with minor modifications. Briefly, cells (1×10^4^ cells/well) were seeded in a 96-well clear bottom black plate and incubated overnight. Ze-IR700 (4 μM) was added into the cells and further incubated for 0.5 h at 37 °C. After two washes with DMEM supplemented with 10% fetal bovine serum (FBS), the cells were irradiated by a laser with a fluence rate of 16 mW/cm^2^ for a total dose of 10 J/cm^2^ at 690 nm. The surviving cells were measured on the next day using CCK-8. To visualize the live and dead cells, approximately 2×10^4^ cells were inoculated in the 96-well plate and treated with PDT. SYTO 9 (indicating live cells) and propidium iodide (PI, indicating dead cells) were added to the cells according to the instructions of the Live/Dead BacLight Bacterial Viability Kit (Invitrogen, CA) at 1 h post-PDT. To verify the role of Ze as a carrier for IR700, the Ze affibody in Ze-IR700 was disrupted by digestion Ze-IR700 with trypsin. The Ze-IR700 digested with trypsin (Ze-IR700-TN) was used as a control in some experiments.

To define the target organelle for Ze-IR700-mediated PDT in CRC cells, the accumulation of Ze-IR700 in mitochondria and lysosomes were observed under a multiphoton laser confocal microscope. Cells (2×10 ^5^ cells in 300 μL medium) were incubated with the mixture of Ze-IR700 (5 μM), organelle tracker (MitoTracker for mitochondria, LysoTracker for lysosomes; 200 nM; Thermo, MA), and Hoechst (indicating nuclei) at 37 °C for 0.5 h. To determine the involvement of ROS in Ze-IR700-mediated PDT, dichlorodihydrofluorescein diacetate (DCFH, Beyotime, Shanghai, China) and singlet oxygen sensor green (SOSG, Invitrogen, Carlsbad, CA) were used to detect ROS in irradiated cells according to the descriptions by Shi *et al.* 29. Ze-IR700 digested with trypsin (Ze-IR700-TN) was used as a control for Ze-IR700.

To determine the expression of DR4 and DR5 in CRC cells by flow cytometry, 4×10^5^ cells (in 100 μL medium) were incubated with 10 µM (for LS174T) or 5 µM (for HT29) Ze-IR700 in an Eppendorf tube at 37 °C for 0.5 h. After two washes with PBS, the cells were transferred to a 48-well plate and irradiated at 690 nm for a total dose of 10 J/cm^2^. Subsequently, these cells were incubated with the primary antibodies against DR4 or DR5 for 1 h followed by incubation with corresponding secondary antibodies for 0.5 h prior to flow cytometry analysis. The cells treated without PDT were used as a control.

To investigate the impact of Ze-IR700-mediated PDT on the gene expression of CRC cells by RNA sequencing, 5×10^5^ cells were incubated with 1 μM Ze-IR700 in an Eppendorf tube containing 500 μL of medium at 37 °C for 0.5 h. After two washes with PBS, the cells were transferred to a 48-well plate for irradiation (690 nm, 10 J/cm^2^). These cells were collected at 1 h or 24 h postirradiation for total RNA extraction using TRIzol regent according to the manual. RNA sequencing was performed by Beijing Genomics Institution (Shenzhen, China). Cells not subjected to PDT treatment were used as a control.

### Flow cytometry and immunofluorescence

The expression levels of EGFR, DR4, DR5, CD31, and hypoxia inducible factor-1α (HIF1α), cleaved caspase 3, cleaved PARP, and Ki67 were examined using flow cytometry or immunofluorescence with primary antibodies and their corresponding secondary antibodies. The primary antibodies include rabbit anti-human EGFR, DR4, DR5, HIF1α (Abcam, MN), Ki67 (Invitrogen, CA), cleaved PARP, or cleaved caspase 3 (ZEN Bioscience, Chengdu, China), and rat anti-mouse CD31 (BioLegend, CA). The secondary antibodies derived from donkeys or goats were labeled with DyLight 550 or DyLight 488 (Abcam, MN). The primary antibodies were incubated with tumor cells or tumor tissues at room temperature for 1.5 h prior to incubation with the secondary antibody for additional 0.5 h.

To determine the binding of protein, 3×10^5^ cells were incubated with 200 nM FAM- or 4 μM IR700-labeled Ze affibody at 4 °C for 1 h prior to analysis on the flow cytometer (Cytomics FC500, Beckman, CA). All data were analyzed using FLOWJO software.

### Western blot

To measure the regulation of death receptors (DR4 and DR5) and decoy receptors (DcR1 and DcR2) by PDT, 4×10^5^ LS174T cells were incubated with Ze-IR700 (2 μM) at 37 °C for 0.5 h followed by irradiation. To examine the downregulation of death receptors by siRNA, 6×10^4^ HCT116 cells were incubated with 75 nM siRNA specific for DR4 (siDR4, 5'-CCACAAAGAAUCAGGCAAU-3') or DR5 (siDR5, 5'-CAGCCGUAGUCUUGAUUGU-3') for 48 h in 24-well plate. Irrelevant siRNA (siNC, 5'-UUCUCCGAACGUGUCACGU-3') was used as a negative control. The membrane proteins of cells were extracted using RIPA lysis buffer (Beyotime, Shanghai, China) for SDS-PAGE. Western blot was performed with rabbit anti-human DR4, DR5, DcR1, (Abcam, MN), cleaved PARP, cleaved caspase-3, caspase-9 (ZEN Bioscience, Chengdu, China), and mouse anti-human cleaved caspase-8 (Cell Signaling Technology, MA). To eliminate ROS, 4 mM antioxidant Acetylcysteine (NAC) (Selleck Chemicals, TX) was added into cells before PDT.

### Tumor graft animal model and treatment

All applicable institutional guidelines for the care and use of animals were followed. CRC cells (2×10^6^ cells for COLO 205 and LS174T, 5×10^6^ cells for HT29, in 100 μL PBS) were subcutaneously injected into the right hind legs of 4- to 6-week-old female BALB/c nude mice. The longitudinal (L) and transverse (W) diameters of the tumor grafts were measured, and the tumor volume (V) was calculated according to the formula: V = L×W×W/2. The mice bearing tumor grafts were treated with TRAIL proteins or Ze-IR700-mediated PDT as a monotherapy or combination therapy. After treatment, the tumor volume was measured every day. Once the tumor volume reached 1000 mm^3^, the mice were sacrificed. In some experiments, to monitor the tumor growth by optical imaging, tumor graft animal models were constructed by subcutaneous injection of red fluorescent protein (RFP)-expressing CRC cells (produced by Ji Manchu Biotechnology, Shanghai, China).

To evaluate the antitumor effect of TRAIL proteins as a monotherapy, mice bearing tumor grafts were intravenously injected with a single dose of IgBD-TRAIL (3 mg/kg for COLO 205, 10 mg/kg for LS174T and HT 29) or TRAIL (10 mg/kg). PBS was used as a control.

To evaluate the antitumor effect of Ze-IR700-mediated PDT as a monotherapy, mice bearing tumor grafts were intravenously injected with Ze-IR700 (1 or 3 mg/kg). Irradiation (fluence rate 100 mW/cm^2^ for a total dose 60 J/cm^2^) was performed 4 h postinjection of Ze-IR700. The mice in the control group were injected with the same volume of PBS. To examine the tissue damage mediated by PDT, tumor grafts were collected at different time post-PDT, and the histology of tumor tissues was examined by H&E staining.

To evaluate the antitumor effect of combination therapy with TRAIL proteins and Ze-IR700-mediated PDT, mice were intravenously injected with 1 mg/kg Ze-IR700. IgBD-TRAIL or TRAIL (3 mg/kg) was intravenously injected into mice immediately prior to irradiation (fluence rate 100 mW/cm^2^ for a total dose 60 J/cm^2^) of the mice. Monotherapy with PDT, IgBD-TRAIL, and TRAIL was performed simultaneously. To compare the apoptosis induction abilities of these therapies, tumor grafts were collected at 24 h posttreatment for apoptotic cell detection using terminal deoxynucleotidyl transferase dUTP nick end labeling (TUNEL) assay (Promega, WI), immunofluorescence with antibody against cleaved caspase 3 or cleaved PARP. The proliferative cells in tumor grafts were illustrated using Ki67 as indicator. Simultaneously, the tissue damage in these tumor grafts and normal tissues (liver and kidney) was illustrated by H&E staining.

### Statistical analysis

One-way analysis of variance (ANOVA) for multiple comparisons was performed using SPSS software version 17.0. The results are expressed as the mean ± standard error (SEM), and the significance level was defined as *p <* 0.05.

## Results

### Monotherapy with long-acting TRAIL eradicates large tumor grafts of CRC cells with chemotherapeutic MDR, but not CRC cells with both chemotherapeutic MDR and TRAIL resistance

**Figure 1:**
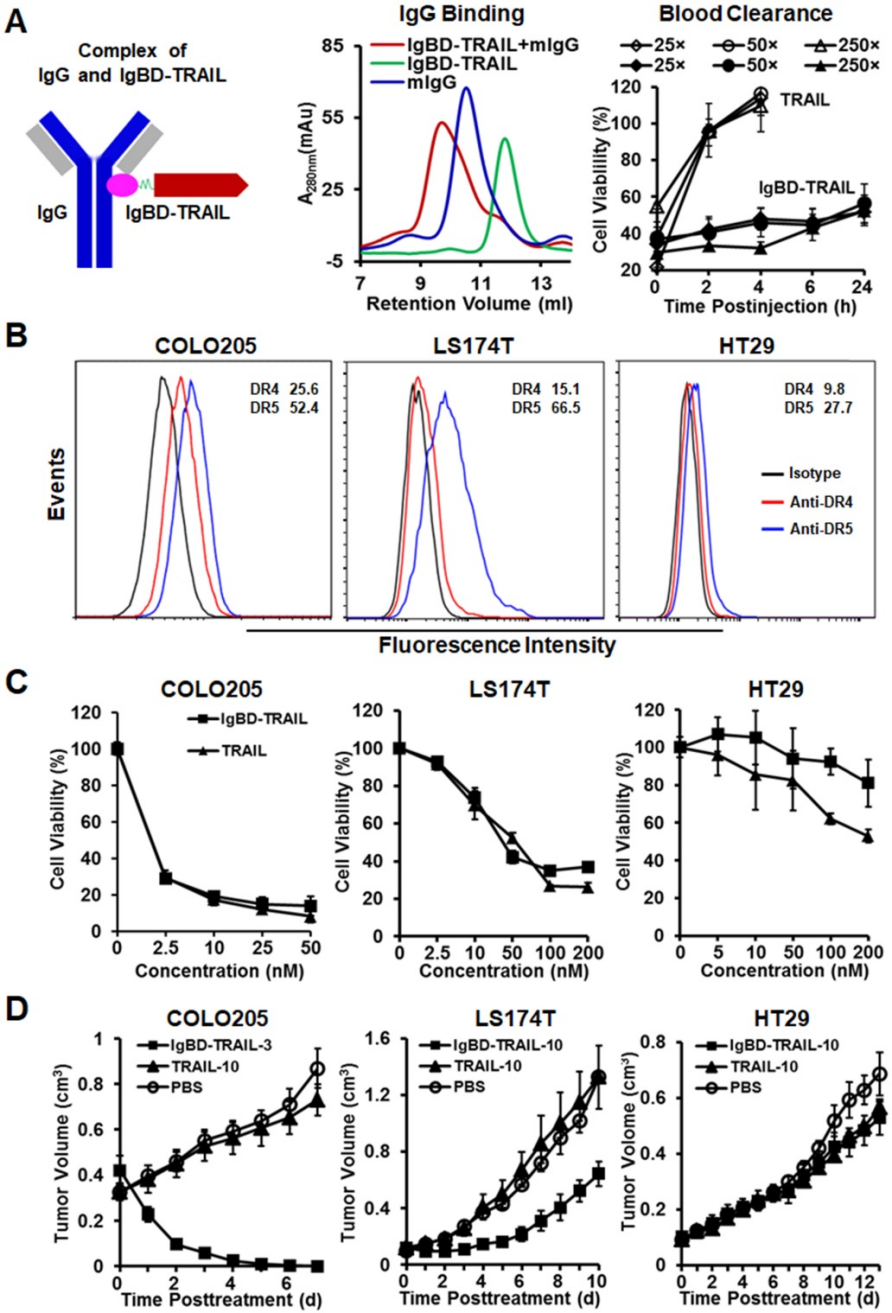
** Antitumor effect of TRAIL proteins in mice bearing tumor grafts of CRC cells. A.** Characterization of TRAIL proteins. IgBD-TRAIL was produced by fusing the IgG-binding domain (IgBD) to the N-terminus of TRAIL. Binding of IgBD-TRAIL to mIgG was analyzed by SEC. The difference in blood clearance of IgBD-TRAIL and TRAIL was reflected by the time-dependent reduction in cell death induced by the residual proteins in plasma (diluted 25, 50 and 250 times). **B.** Expression of DR4 and DR5. The percentages of the death receptor-positive cells are indicated. **C.** Cytotoxicity of TRAIL proteins. **D.** Antitumor effect of a single dose of TRAIL proteins administered at day 0 in mice (N=5-6) bearing tumor grafts of COLO 205, LS174T or HT29 cells. COLO 205 tumor grafts were treated with 3 mg/kg IgBD-TRAIL (IgBD-TRAIL-3) or 10 mg/kg TRAIL (TRAIL-10). The mice bearing LS174T or HT29 tumor grafts were treated with 10 mg/kg of TRAIL proteins.

The long-acting TRAIL, *i.e.*, IgBD-TRAIL, was constructed by fusing an IgG-binding domain (IgBD) to the N-terminus of TRAIL, which allowed IgBD-TRAIL to bind IgG (Figure 1A). According to our previous works 28, as IgG exhibits a long circulation in blood, the serum half-life of IgG-binding IgBD-TRAIL was extended to 50-60 times longer than that of TRAIL, thus showing a greater antitumor effect. Here, before evaluation on the synergy between IgBD-TRAIL and PDT, we verified the IgG-binding ability and long circulation of IgBD-TRAIL. After incubation of IgBD-TRAIL with mIgG, SEC analysis demonstrated a novel protein peak with an apparent molecular weight that was definitely higher than that of individual mIgG (Figure 1A), indicating that IgBD-TRAIL could bind IgG. To compare the blood clearance, plasma samples of mice injected with a single dose of IgBD-TRAIL or TRAIL were collected at different time points for cytotoxicity assay. As shown in Figure 1A, cytotoxicity of the blood samples from mice injected with TRAIL was undetectable from 2 h postinjection. However, the cytotoxicity of blood samples from mice injected with IgBD-TRAIL persisted at least 24 h. These results indicated that IgBD-TRAIL could bind endogenous IgG, thus showing significantly prolonged serum half-life.

CRC cells, including COLO 205, COLO 320 DR, COLO 320 HSR, LS174T, HT29, HCT-8, HCT116, LOVO, RKO, T84, and LS180, are resistant to multiple chemical drugs, such as cisplatin, vincristine, doxorubicin, and bortezomib (Figure S1A), indicating the chemotherapeutic MDR is common in CRC cells. Due to the expression of surface death receptor DR4 and DR5 (Figure 1B), TRAIL showed cytotoxicity in these CRC cells, which might overcome chemotherapeutic MDR of CRC cells. As shown in Figure 1C and Figure S1B, TRAIL exerted potent cytotoxicity in COLO 205 and HCT116 cells. Nevertheless, the IC50s of TRAIL in LS174T and LS180 (30-40 nM), HT29 and LOVO (200-300 nM), and other CRC cells (> 500 nM) were much higher than those of TRAIL in COLO 205 and HCT116 cells (0.2-0.5 nM). Consequently, in this paper, COLO 205 and HCT116 cells with chemotherapeutic MDR were defined as TRAIL-sensitive cells. The other CRC cells with chemotherapeutic MDR were defined as TRAIL-resistant cells. Although the *in vitro* cytotoxicity of TRAIL was similar to that of IgBD-TRAIL in COLO 205 cells (Figure 1C), intravenous injection of a single low-dose (3 mg/kg) IgBD-TRAIL, but not high-dose (10 mg/kg) TRAIL, eradicated large (~300 mm^3^) tumor grafts of COLO 205 cells within 1 week. Tumor uptake analysis demonstrated that IgBD-TRAIL with long circulation accumulated more than TRAIL in tumor grafts (Figure S2), contributing to the greater antitumor effect of IgBD-TRAIL. Unexpectedly, IgBD-TRAIL only showed mild to moderate growth suppression on HT29 and LS174T tumor grafts, suggesting the need to combine IgBD-TRAIL with sensitizers to combat these CRC cells with both chemotherapeutic MDR and TRAIL-resistance.

### Monotherapy of tumor cell-targeted PDT effectively induces death of CRC cells with chemotherapeutic MDR and TRAIL resistance but cannot eradicate their tumor grafts

**Figure 2:**
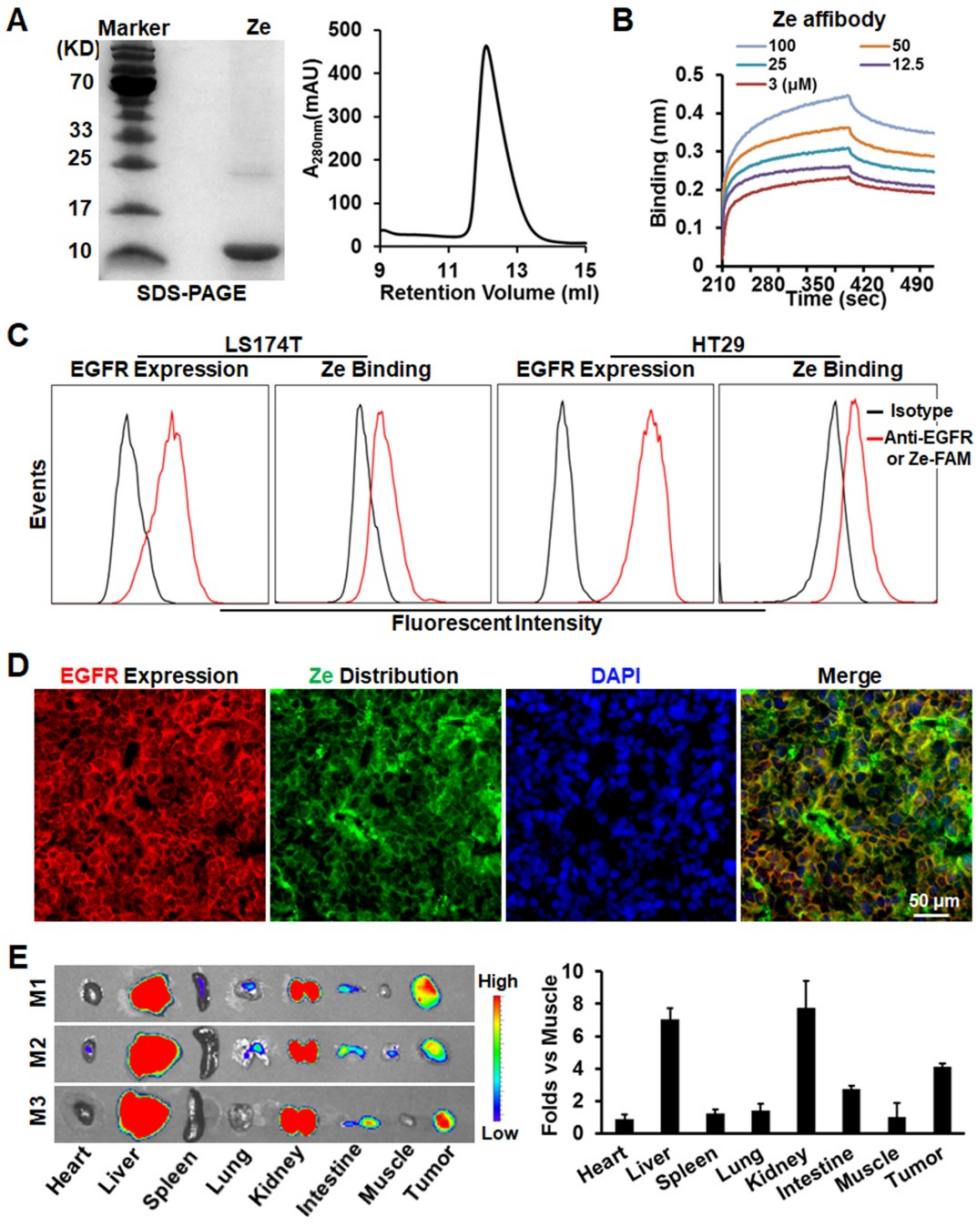
** Tumor-homing characteristics of Ze affibody with high affinity for EGFR. A.** SDS-PAGE and SEC analysis of purified Ze affibody. **B.** Binding of Ze affibody to EGFR analyzed by biolayer interfereometry. **C.** EGFR expression in LS174T and HT29 cells and binding of Ze affibody to these cells. **D.** Cellular distribution of Ze affibody in LS174T tumor grafts. **E.** Tissue distribution of Ze affibody in mice bearing LS174T tumor grafts. Organs/tissues derived from mice (N=3) were scanned by an IVIS optical imaging system at 4 h postinjection of CF750-labeled Ze affibody.

To perform tumor cell-targeted PDT, EGFR-specific Z_EGFR_ (Ze) affibody was used as a carrier for IR700 photosensitizer. As shown in Figure 2A, the Ze affibody recovered from *E. coli* appeared as a single protein band on SDS-PAGE gel and a single protein peak on an SEC column, indicating that the Ze affibody was purified to homogeneity. Protein-protein interaction assay revealed that the Ze affibody bound EGFR with a subnanomolar affinity (KD=0.43±0.57 nM, Figure 2B). Flow cytometry analysis demonstrated that the Ze affibody could bind EGFR-expressing LS174T and HT29 cells (Figure 2C). Moreover, intravenously injected Ze affibody was predominantly distributed on EGFR-expressing cells (Figure 2D, Figure S3A), thus accumulating in LS174T tumor grafts in mice (Figure 2E). In contrast, Ze affibody digested with trypsin (Ze-TN) was undetectable in tumor grafts (Figure S3B, C). These results demonstrated that the Ze affibody could accumulate in tumor grafts of EGFR-expressing CRC cells, indicating the potential of the Ze affibody as a carrier for photosensitizer.

**Figure 3:**
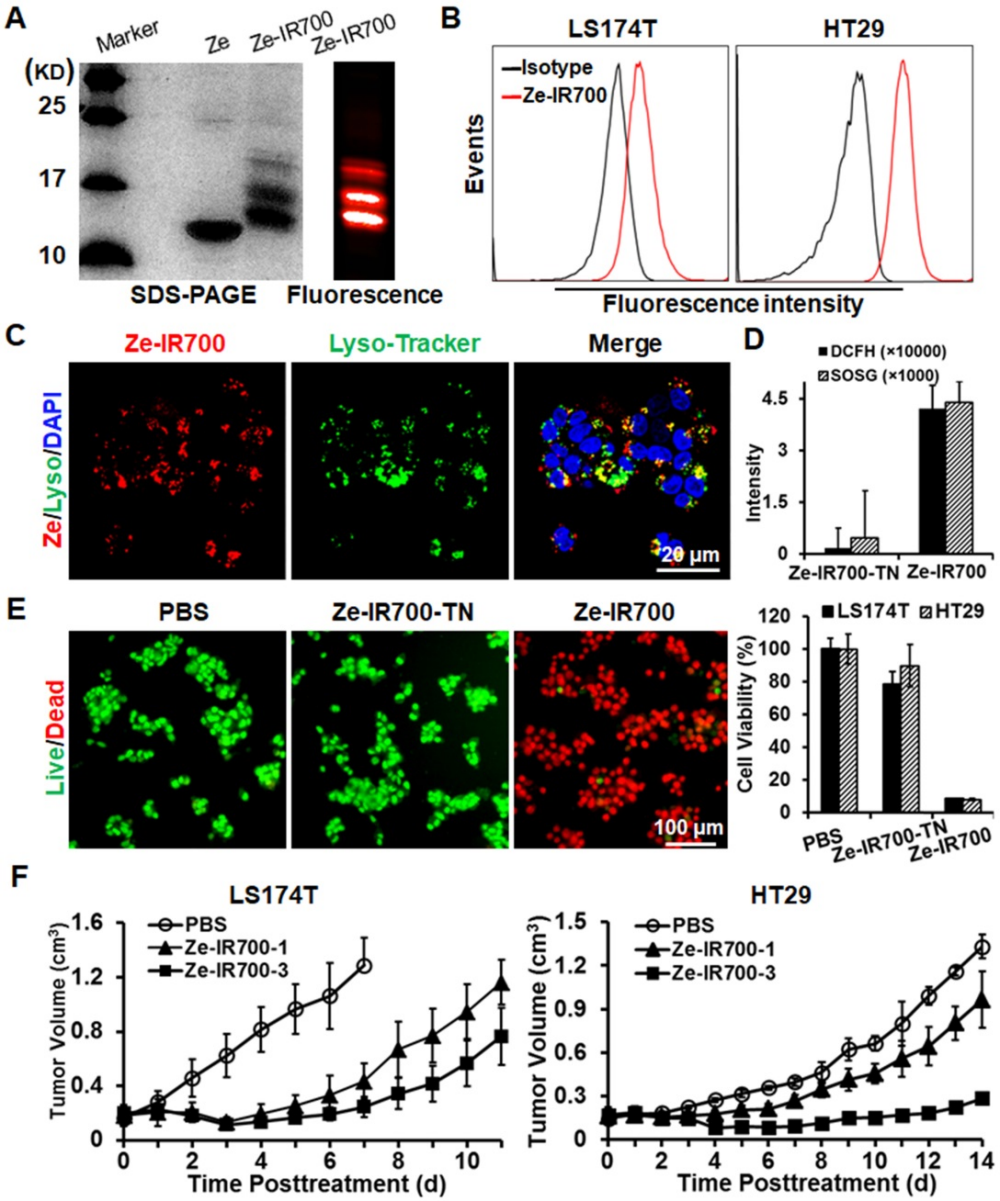
** Antitumor effect of Ze-IR700-mediated PDT in mice bearing tumor grafts of CRC cells. A.** Preparation of Ze-IR700 by conjugating the photosensor IR700 to Ze affibody. **B.** Binding of Ze-IR700 to LS174T and HT29 cells. **C.** Accumulation of Ze-IR700 in the lysosomes of LS174T cells. **D.** ROS produced in LS174T cells treated with Ze-IR700- or trypsinized Ze-IR700 (Ze-IR700-TN)-mediated PDT. **E.** Death of LS174T and HT29 cells treated with Ze-IR700- or Ze-IR700-TN-mediated PDT. **F.** Tumor growth suppression by Ze-IR700-mediated PDT. Mice (N=5) bearing LS174T or HT29 tumor grafts were intravenously injected with 1 mg/kg (Ze-IR700-1) or 3 mg/kg (Ze-IR700-3) Ze-IR700 followed by irradiation 4 h later.

Subsequently, the photosensitizer IR700 was conjugated to the Ze affibody to produce EGFR-targeted Ze-IR700 (Figure 3A). Flow cytometry analysis demonstrated that Ze-IR700 could bind LS174T and HT29 cells (Figure 3B). After incubation with cells at 37 °C for 0.5 h, Ze-IR700 could enter LS174T cells and was predominantly distributed in lysosomes (Figure 3C), but not in mitochondria (Figure S4). To determine the cell death induced by PDT, the CRC cells were incubated with Ze-IR700 or trypsinized Ze-IR700 (Ze-IR700-TN) followed by two washes with medium prior to irradiation. DCFH and SOSG assays demonstrated that Ze-IR700, but not Ze-IR700-TN-mediated PDT, produced high levels of ROS (Figure 3D). Accordingly, Ze-IR700-mediated PDT induced over 90% cell death in LS174T and HT29 cells. In contrast, the same amount of Ze-IR700-TN-mediated PDT showed little cytotoxicity in both cell types (Figure 3E and Figure S5A). In mice bearing large (~150 mm^3^) tumor grafts of LS174T or HT29 cells, PDT exerted Ze-IR700 dose-dependent tumor growth suppression. As shown in Figure 3F, 1 mg/kg Ze-IR700-mediated PDT showed obvious tumor growth suppression. When the amount of Ze-IR700 was increased from 1 mg/kg (Ze-IR700-1) to 3 mg/kg (Ze-IR700-3), the tumor growth suppression of PDT was further enhanced. However, both 1 and 3 mg/kg Ze-IR700-mediated PDT did not eradicate any tumor grafts in these mice. These results demonstrated that monotherapy of Ze-IR700-mediated PDT exerted potent tumor growth suppression but did not eradicate tumor grafts of these CRC cells with chemotherapeutic MDR and TRAIL resistance.

### Tumor cell-targeted PDT sensitizes CRC cells to TRAIL by upregulating death receptors and downregulating decoy receptors

**Figure 4:**
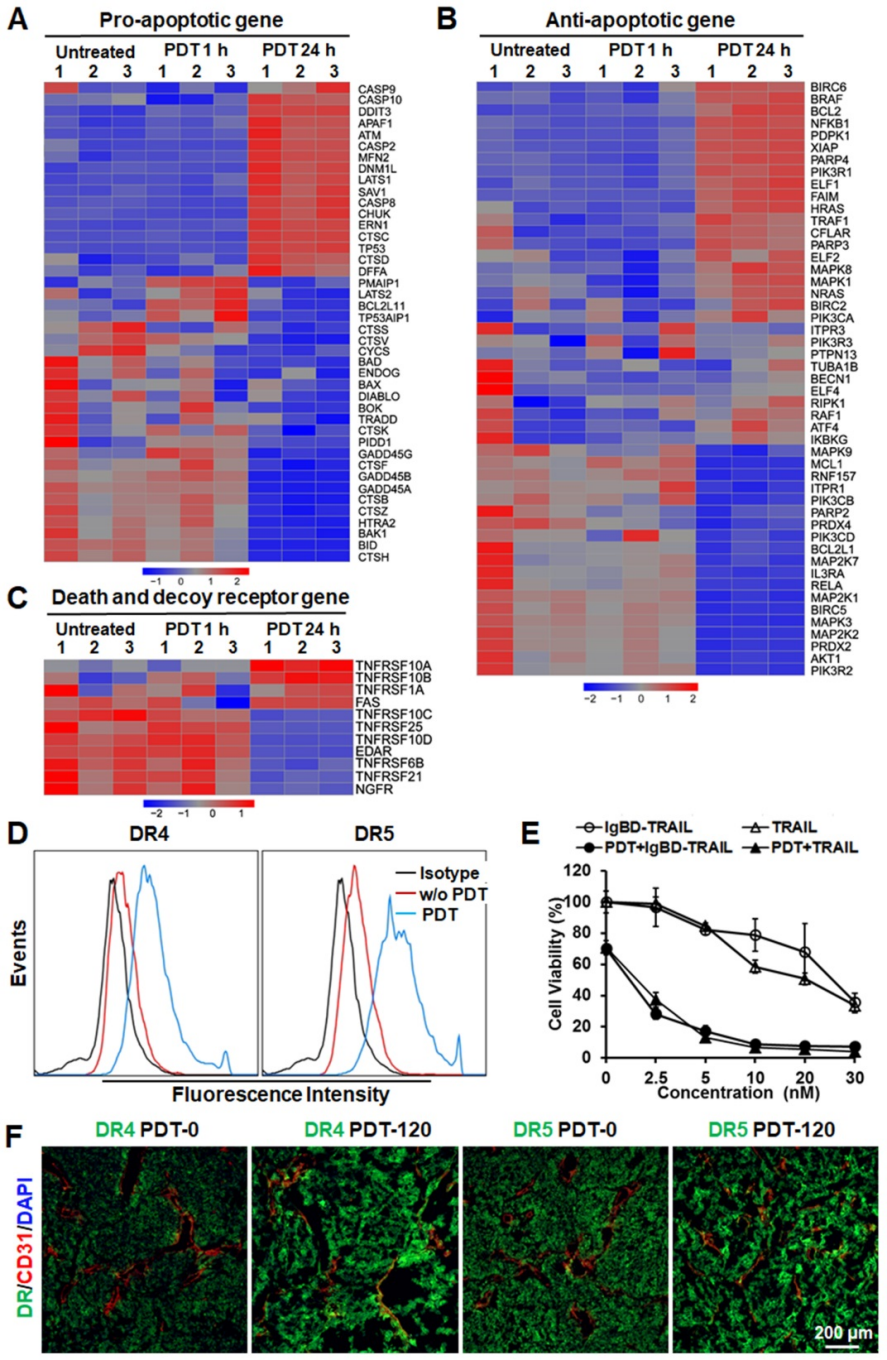
** Ze-IR700-mediated PDT upregulated TRAIL receptors and exerted synergistic cytotoxicity with TRAIL proteins in LS174T cells. A,B,C.** Expression profiles of pro-apototic genes (A), anti-apoptotic genes (B), and death receptors and decoy receptors (C) in LS174T cells treated with or without PDT. **D.** Flow cytometry analysis of DR4 and DR5 in LS174T cells treated with or without PDT.** E**. Cytotoxicity of TRAIL proteins in LS174T cells treated with or without PDT.** F.** Expression profile of DR4 and DR5 in tumor tissues treated with or without PDT. The tumor tissues collected before PDT (PDT-0) or at 2 h post-PDT (PDT-120) were frozenly sectioned and stained with antibody against DR4 or DR5. Tumor microvessels were illustrated by CD31 staining. The nuclei were visualized by DAPI staining.

Cell death analysis demonstrated that Ze-IR700-mediated PDT induced caspase-dependent apoptosis in LS174T cells (Figure S5B, C). RNA sequencing analysis revealed that the mitochondria-associated pro-apoptotic proteins, including Bid, BAD, BAK1, BOK, and BAX, were downregulated in LS174T cells by sublethal PDT. However, PDT induced upregulation of pro-apoptotic proteins, such as caspases (CSCP 2, 8), APAF1, DDIT3, cathepsins (CTSC, CTSD), TP53, ATM, and DFFA (Figure 4A, and Table S1), suggesting that PDT predominantly induced lysosome-associated apoptosis. In addition to upregulating pro-apoptotic factors, Ze-IR700-mediated PDT also increased the expression of anti-apoptotic genes such as BCL2, BCL2L1, BIRC2 and BIRC6, XIAP, CFLAR, FAIM, HRAS and NRAS, ELF family (ELF1, 2, 4), PARP family (PARP2, 4), PIK3CA, PIK3R1 and PIK3R3 (Figure 4B and Table S2), indicating the potential to enhance the antitumor effect of PDT by combination with other apoptosis inducer.

Notably, RNA sequencing analysis revealed that Ze-IR700-mediated PDT significantly (*p <* 0.01) increased the expression of membrane TRAIL receptors, including death receptor DR4 (TNFRSF10A) and DR5 (TNFRSF10B). However, TRAIL decoy receptors, including DcR1 (TNFRSF10C) and DcR2 (TNFRSF10D), were down-regulated (Figure 4C and Table S3), suggesting the effective synergy between PDT and TRAIL in inducing apoptosis of CRC cells. Flow cytometry analysis verified the upregulation of DR4 and DR5 in LS174T cells after PDT treatment. As shown in Figure 4D, the positive rates of death receptors in LS174T treated with Ze-IR700-mediated PDT were 83.9% (DR4) and 96.5% (DR5), compared to 16.5% (DR4) and 27.4% (DR5) of that in the untreated LS174T cells. Further western blot revealed that PDT-induced upregulation of DR4 and DR5 and downregulation of DcR1 in LS174T cells. Addition of antioxidant NAC into the cells abolished the impact of PDT on expression of death receptor and decoy receptor in these cells (Figure S6A), indicating that PDT upregulated TRAIL receptors by ROS. Accordingly, the sensitivity of LS174T cells to TRAIL proteins was drastically increased by PDT. The IC50s of IgBD-TRAIL and TRAIL in LS174T cells treated with PDT were over 10-40 times (approximately 1-3 nM vs 30-40 nM) lower than that in LS174T cells without PDT treatment (Figure 4E), demonstrating the synergy between PDT and TRAIL proteins in killing CRC cells. Addition of soluble DR4-Fc or DR5-Fc into the in PDT-treated CRC cells abrogated the synergy (Figure S6B), indicating that the synergy between PDT and TRAIL protein in killing CRC cells is death receptor-dependent. Moreover, the expression of DR4 and DR5 in tumor tissues definitely increased after PDT treatment (Figure 4F), suggesting that the Ze-IR700-mediated PDT would sensitize tumor cells to TRAIL protein under *in vivo* condition. Although HT29 cells exerted greater chemotherapeutic MDR and TRAIL-resistance than did by LS174T cells, *in vitro* Ze-IR700-mediated PDT also upregulated the membrane DR4 (from 13.6% to 29%) and DR5 (from 20.6% to 41.1%) in HT29 cells (Figure S7A). Accordingly, the IC50s of TRAIL proteins in HT29 cells treated with PDT were approximately 20-60 times (5-10 nM vs 200-300 nM) lower than those in HT29 cells without PDT treatment (Figure S7B), indicating that PDT also drastically sensitized HT29 cells to TRAIL proteins by upregulating death receptors. Moreover, pretreatment with Ze-IR700-mediated PDT increased the cytotoxicity of TRAIL in COLO 320 DR, COLO 320 HSR, HCT-8, LOVO, RKO, LS180, T84, and HCT116 cells (Figure S7C), indicating that tumor cell-targeted PDT extensively sensitized CRC cells to TRAIL. The fact that downregulation of either DR4 or DR5 abrogated the synergy between PDT and TRAIL in killing HCT116 cells suggested that PDT sensitized other CRC cells to TRAIL by increase the expression of death receptors (Figure S8).

### Combination therapy of IgBD-TRAIL and tumor cell-targeted PDT efficiently eradicates large tumor grafts of CRC cells with chemotherapeutic MDR and TRAIL resistance

**Figure 5:**
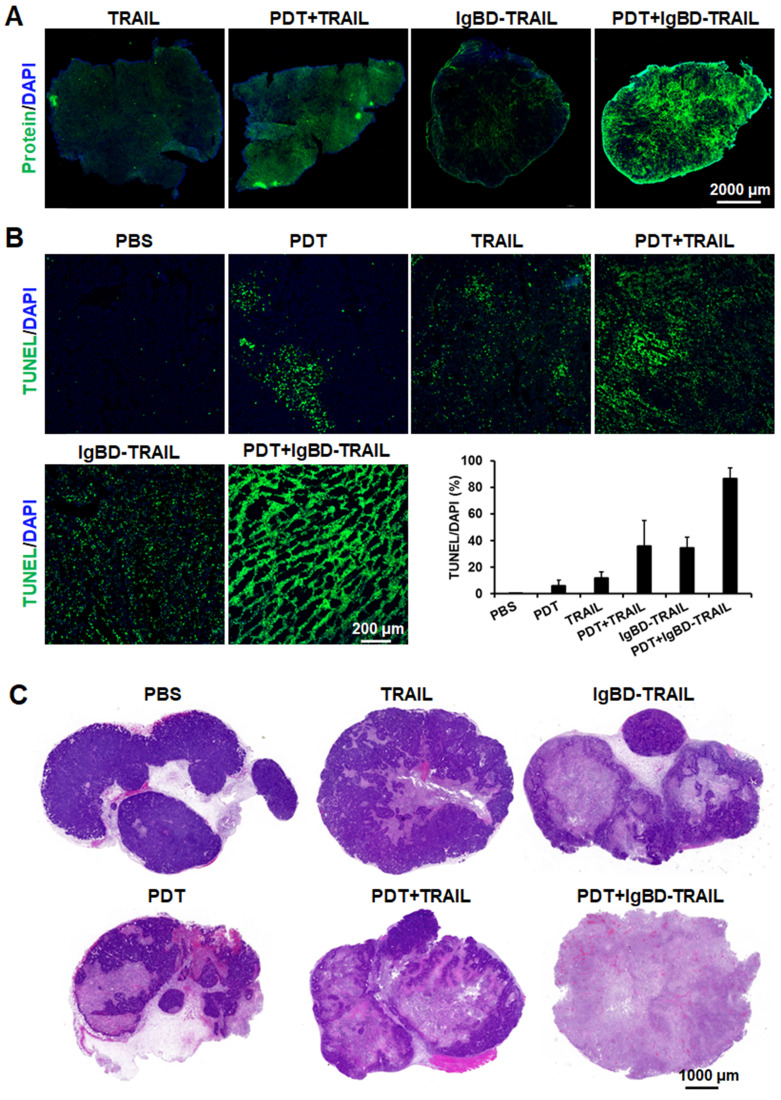
** Ze-IR700-mediated PDT increased tumor uptake and apoptosis induction of TRAIL proteins in LS174T tumor grafts. A.** Uptake of TRAIL proteins by tumor grafts treated with or without PDT.** B.** Apoptosis induction of TRAIL proteins in tumor grafts treated with or without PDT. The tumor tissues were collected 24 h after treatment, and the apoptotic cells were visualized by TUNEL staining. The nuclei were illustrated by DAPI staining. The apoptotic rate was expressed as the ratio of TUNEL-positive cells (apoptotic cells) to the DAPI-positive cells (total cells). **C.** Histology of tumor grafts treated with monotherapy or combination therapy of TRAIL proteins and PDT. The tumor tissues were collected 24 h after treatment and stained with H&E.

In addition to sensitize CRC cells to TRAIL, Ze-IR700-mediated *in vivo* PDT also disrupted the vascular systems of tumor grafts. As shown in Figure S9A, numerous red blood cells and blood clots were observed in LS174T tumor grafts collected at 2 h (PDT-120) and 6 h (PDT-360) post-PDT. Accordingly, expression of HIF1α in tumor tissues increased with time after PDT (Figure S9B), indicating that PDT disrupted tumor vessels and induced hemorrhage and thrombosis, which further intensified hypoxia in tumor grafts. Due to the disruption of tumor vessels, uptake of TRAIL proteins by LS174T tumor grafts was significantly increased (Figure 5A). Notably, the tumor uptake of long-acting IgBD-TRAIL was greater than that of TRAIL with short serum half-life.

Subsequent apoptosis induction assays demonstrated that the combination of TRAIL proteins and Ze-IR700-mediated PDT induced greater apoptosis in LS174T tumor grafts than the monotherapy of either TRAIL proteins or PDT. As shown in Figure 5B, the percentage of apoptotic cells in LS174T tumor grafts treated with 1 mg/kg Ze-IR700-mediated PDT was 6.1±4.2%. Apoptotic cell rates in tumor grafts treated with monotherapy of TRAIL and IgBD-TRAIL were 11.9±4.4% and 34.7±7.9%, respectively. However, the apoptotic cell rates in tumor grafts treated with TRAIL/PDT and IgBD-TRAIL/PDT were 35.8±19.3% and 86.5±8.1%, respectively, which are much higher than their monotherapies. Accordingly, combination therapy of IgBD-TRAIL and PDT induced greater activation of caspases *in vitro* (Figure S10A) and *in vivo* (Figure S10B). The proliferative cells in tumor grafts treated with combination therapy was definitely less than that in tumor grafts treated with monotherapy (Figure S10B). Further histochemistry analysis demonstrated that monotherapy of Ze-IR700-mediated PDT, TRAIL, or IgBD-TRAIL only partially damaged tumor tissues. Although combination therapy with TRAIL and PDT extensively damaged tumor grafts, many tumor tissues were still visible at the rims of tumor grafts. However, combination of IgBD-TRAIL and Ze-IR700-mediated PDT damaged most tumor tissues in LS174T tumor grafts (Figure 5C). These results indicated that tumor cell-targeted PDT enhanced the *in vivo* caspase-dependent apoptosis induction of TRAIL proteins.

**Figure 6:**
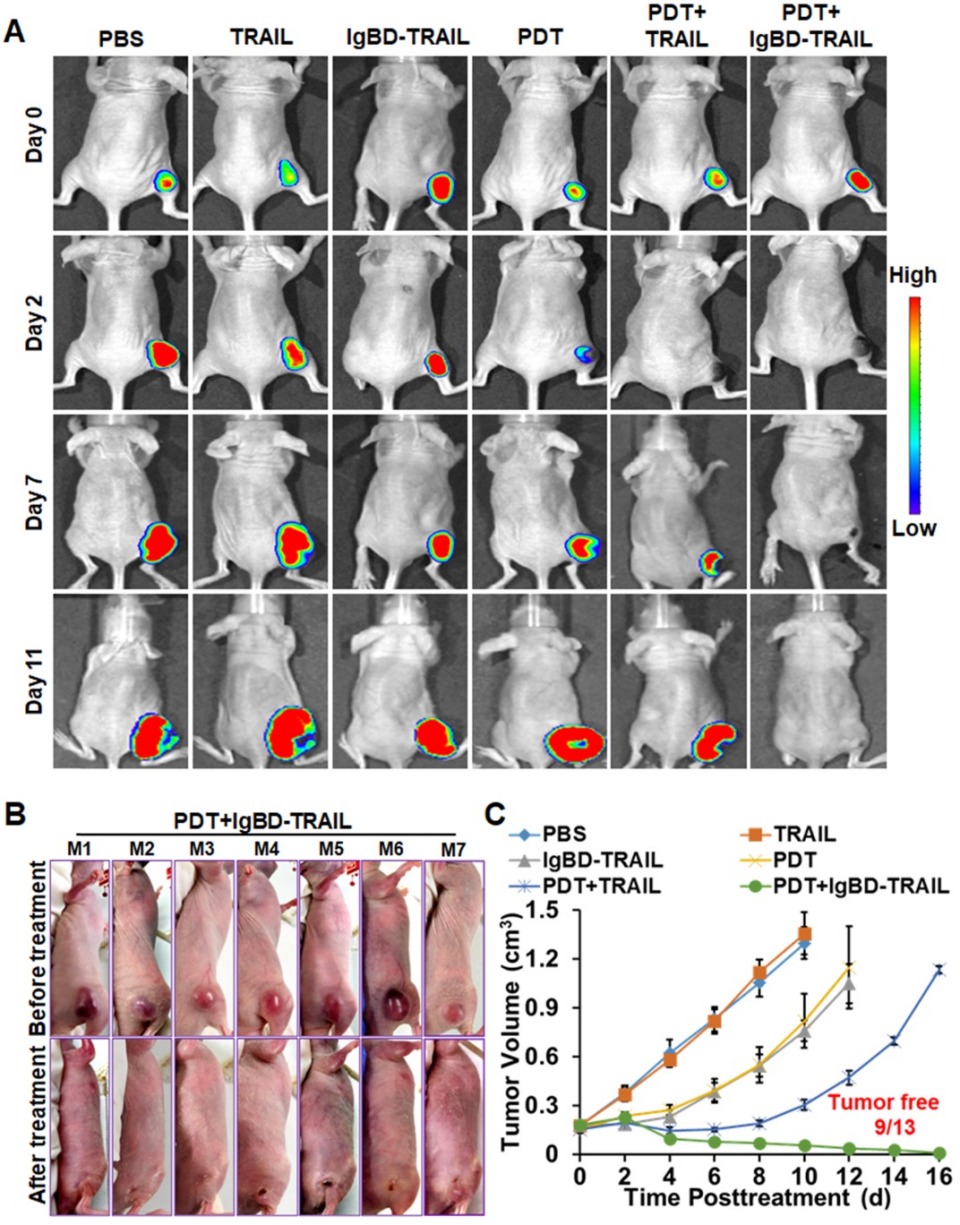
** Antitumor effect of combination therapy of TRAIL proteins and Ze-IR700-mediated PDT in mice bearing LS174T tumor grafts. A.** Dynamic optical imaging of mice bearing tumor grafts of RFP-expressing LS174T cells treated with TRAIL proteins in combination with or without PDT. **B.** Representative pictures of mice bearing tumor grafts before and after treatment with combination therapy of IgBD-TRAIL and PDT. **C.** Growth curves of tumor grafts after treatment with monotherapy or combination therapy of TRAIL proteins and PDT. A single treatment was administered at day 0. The number of mice with tumor eradication (tumor free) is indicated.

The antitumor effect of TRAIL proteins combined with or without Ze-IR700-mediated PDT was first evaluated in mice bearing tumor grafts (~150 mm^3^) of native LS174T cells or RFP-expressing LS174T cells. As shown in Figure 6A, optical images demonstrated that the fluorescence of tumor grafts increases with time after treatment with TRAIL proteins. After treatment with Ze-IR700-mediated PDT and combination therapy with TRAIL and PDT, fluorescence in most tumor grafts decreased at the early time (2-3 day) posttreatment. However, the fluorescence in all tumor grafts treated with combination therapy of IgBD-TRAIL and PDT was undetectable from the second day after treatment. Tumor growth curves demonstrated that TRAIL monotherapy showed no tumor suppression compared to the PBS control. Monotherapy of Ze-IR700-mediated PDT or IgBD-TRAIL showed moderate tumor growth suppression. Combination therapy of TRAIL and PDT induced a progress-free state within one week after treatment. However, obvious hemorrhage accompanied by scab formation was observed in all tumor grafts treated with combination therapy of IgBD-TRAIL and PDT from the second day after treatment. In subsequent days, these tumor grafts continuously shrank, followed by absorption or shedding from the body (Figure 6B). At the end of this observation, 70% (9 out of 13) of tumor grafts were eradicated (Figure 6B, C and Figure S11A). These results demonstrated that Ze-IR700-mediated PDT and TRAIL proteins exerted a synergistic antitumor effect. However, combination of the long-acting IgBD-TRAIL, but not TRAIL with a short serum half-life, and PDT efficiently eradicated LS174T tumor grafts.

**Figure 7:**
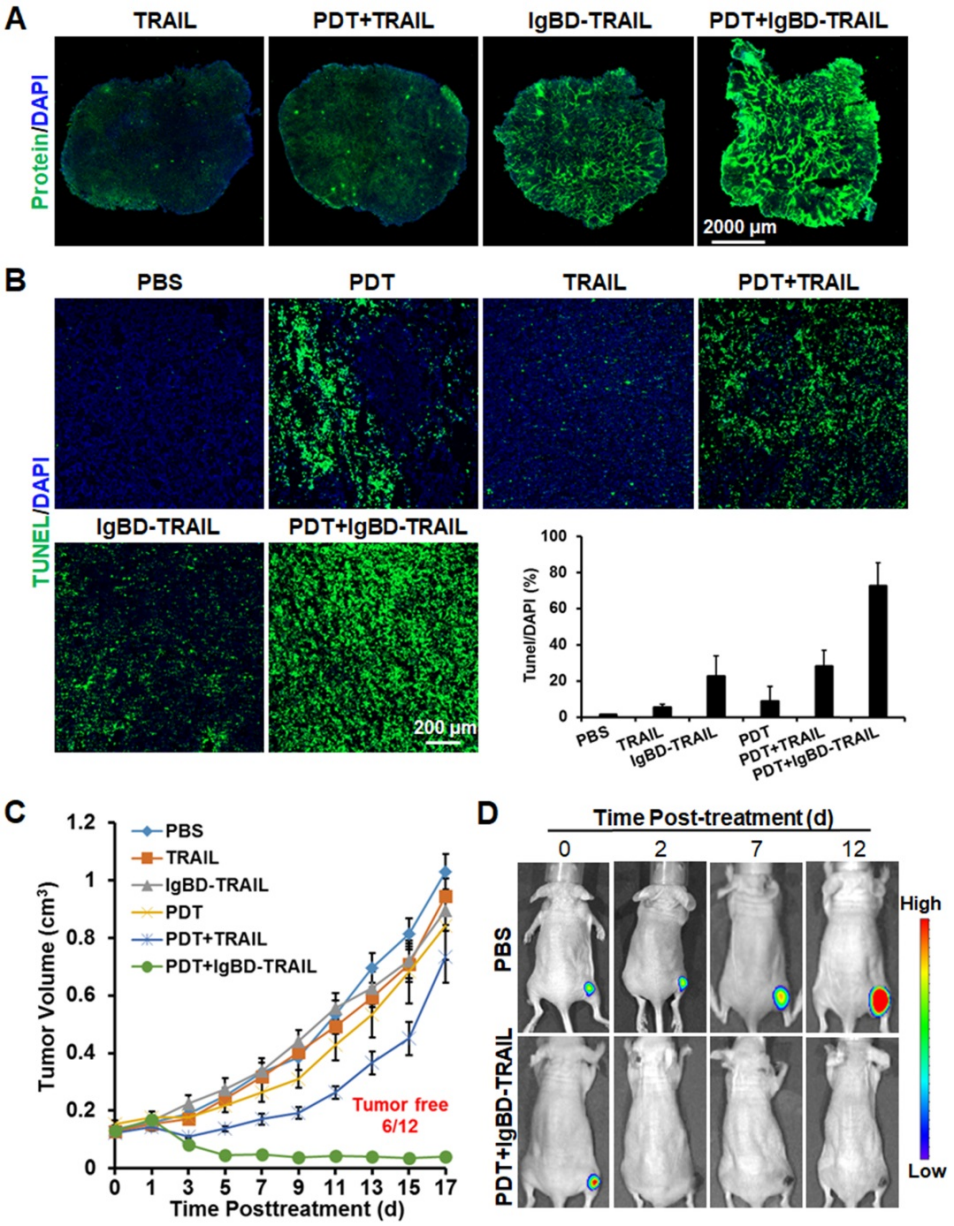
** Antitumor effect of combinational therapy of TRAIL proteins and Ze-IR700-mediated PDT in mice bearing HT29 tumor grafts. A.** Uptake of TRAIL proteins by tumor grafts treated with or without PDT. **B.** Apoptosis induction by monotherapy or combination therapy of TRAIL proteins and PDT in tumor grafts.** C.** Growth curves of tumor grafts treated with monotherapy or combination therapy of TRAIL proteins and PDT. The number of mice with tumor eradication (tumor free) is indicated. **D.** Representative optical images of mice bearing tumor grafts during treatment with combination therapy of IgBD-TRAIL and PDT. A single treatment was administered at day 0.

Similar to that in LS174T tumor grafts, Ze-IR700-mediated PDT also increased the uptake of TRAIL, as did IgBD-TRAIL by HT29 tumor grafts (Figure 7A). Compared to TRAIL with a short serum half-life, the long-acting IgBD-TRAIL combined with Ze-IR700-mediated PDT induced greater (72.6±12.8% vs 28.3±8.7%) apoptosis in HT29 tumor grafts (Figure 7B). Tumor growth curves demonstrated that monotherapy of Ze-IR700-mediated PDT or TRAIL proteins showed little tumor growth suppression. Combination therapy of TRAIL and PDT only regressed the tumor growth within a short period (3-4 days) after treatment. However, combination of IgBD-TRAIL and PDT drastically damaged HT29 tumor grafts, which induced extensive hemorrhage and scab formation from the 2^nd^ or 3^rd^ day posttreatment. Tumor graft absorption or shedding were observed in most mice over the following days. At the end of the observation, 50% (6 out of 12) of tumor grafts were eradicated by combination therapy of IgBD-TRAIL and PDT (Figure 7C, Figure S11B). Optical images of mice bearing tumor grafts of RFP-expressing HT29 cells verified the tumor growth suppression as well as eradication of tumor grafts of the combination therapy of TRAIL proteins and PDT (Figure 7D, Figure S12). These results demonstrated that the combination of long-acting TRAIL, but not TRAIL with short serum half-life is also highly effective against HT29 tumor grafts.

As shown in Figure S13, monotherapy of IgBD-TRAIL and TRAIL in mice bearing tumor grafts did not show obvious reduction in mouse body weight. Nevertheless, TRAIL, especially IgBD-TRAIL combined with PDT induced a slight (~10%) reduction in body weight of mice bearing LS174T tumor grafts within the first 4 days posttreatment. However, histochemistry of the liver and kidney collected from these mice at the end of observation (day 16) did not show obvious abnormality in structure. The body weight decrease mediated by IgBD-TRAIL and PDT combination therapy was less than 5%. These results suggested that the combination therapy of IgBD-TRAIL and PDT might induce slight acute injury in mice.

## Discussion

The high CRC-associated death rate is predominantly attributed to the MDR of CRC cells constitutively expressing ABC transporters to first-line chemotherapeutics^2^, ^4^. In fact, CRC cells are extensively resistant to chemical drugs such as cisplatin, vincristine, doxorubicin, and bortezomib (Figure S1A), indicating that chemotherapeutic MDR is common in CRC cells. As large proteins with different mechanisms might bypass the resistance pathways for small chemicals, TRAIL, with cytotoxicity in chemical-resistant cancer cells, has been considered as a novel therapeutic option for refractory cancers 30. In our previous work, a novel TRAIL variant, *i.e.*, IgBD-TRAIL, was produced by fusing an IgG-binding domain to the N-terminus of TRAIL^28^. Owing to the IgBD-mediated binding to endogenous IgG, IgBD-TRAIL showed a prolonged serum half-life and thus exerted a greater antitumor effect than native TRAIL. In mice bearing TRAIL-sensitive COLO 205 cells, systemic administration of low-dose (3 mg/kg) IgBD-TRAIL eradicated all large (~300 mm^3^) tumor grafts (Figure 1D), which triggered our interest to investigate the potential of IgBD-TRAIL in combating CRC with greater MDR. It was found that LS174T and HT29 CRC cells were more resistant than COLO 205 cells to cisplatin, vincristine, doxorubicin, bortezomib (Figure S1A) and TRAIL (Figure 1C). Accordingly, intravenous injection of high-dose (10 mg/kg) IgBD-TRAIL only showed mild tumor growth suppression in mice bearing LS174T or HT29 tumor grafts (Figure 1D), indicating that monotherapy of TRAIL proteins is hopeful but inefficient to combat CRC cells with both chemotherapeutic MDR and TRAIL resistance. Definitely, it is needed to develop combinational strategies to overcome both chemotherapeutic MDR and TRAIL resistance of CRC cells.

TRAIL induces apoptosis by ligation to death receptors on the surface of cancer cells 31, suggesting the possibility for enhancing the cytotoxicity of TRAIL by increasing membrane DR4 and/or DR5. It was reported that ROS could increase death receptor expression in cancer cells 17, suggesting the synergy between PDT producing ROS 20, 21 and TRAIL in killing CRC cells with both chemotherapeutic MDR and TRAIL resistance. In fact, we found that Ze-IR700-mediated PDT produced ROS and thus induced apoptosis of LS174T cells (Figure 3D and E, Figure S5). Further RNA sequencing analysis demonstrated that a sublethal dose of Ze-IR700-mediated PDT predominantly (*P <* 0.01) induced lysosome-associated pro-apoptotic factors in LS174T cells (Figure 4A, Table S1). Surprisingly, Ze-IR700-mediated PDT also increased a variety of anti-apoptosis and cell proliferation-associated genes (Figure 4B, Table S2), suggesting that the monotherapy of PDT might show a limited antitumor effect. In fact, monotherapy of Ze-IR700-mediated PDT produced tumor growth suppression, but not eradication of any tumor grafts of both LS174T and HT29 cells with chemotherapeutic MDR and TRAIL resistance (Figure 3F).

Similar to Ze-IR700-mediated PDT, TRAIL also induced apoptosis of LS174T cells (Figure S5B, C), suggesting the crosstalk between PDT and TRAIL-mediated cell death pathways. Interestingly, Ze-IR700- mediated PDT significantly enhanced the expression of death receptors for TRAIL (DR4 and DR5) in both LS174T (Figure 4C, 4D, Figure S6A, Table S3) and HT29 cells (Figure S7A), whereas the decoy receptors of TRAIL (DcR1 and DcR2) in LS174T cells were downregulated by PDT (Figure 4C, Figure S6A, Table S3). These results suggested the synergy between TRAIL and PDT in killing CRC cells. In fact, the sensitivity of LS174T and HT29 cells was increased 10-60 times by PDT (Figure 4E, Figure S7B). In addition, Ze-IR700-mediated PDT also sensitized other CRC cells such as COLO 320DM, COLO 320HSR, LOVO, T84, HCT-8, RKO, LS180, and HCT116 cells to TRAIL (Figure S7C). As these CRC cells are also resistant to chemicals (cisplatin, vincristine, doxorubicin, and bortezomib) and TRAIL proteins (TRAIL and IgBD-TRAIL) (Figure S1), extensive sensitization of these cells to TRAIL proteins by PDT suggested the potential of combination therapy of TRAIL proteins and tumor cell-targeted PDT in combating refractory CRC.

According to our results, the serum half-lives of the TRAIL proteins are closely related to the synergistic antitumor effect of these proteins and PDT. On the one hand, TRAIL protein with longer serum half-life might obtain more chances to bind and kill tumor cells sensitized by PDT. As shown in Table S3, both DR4 and DR5 were only slightly induced within 1 h post-PDT, whereas their expression was increased approximately two-fold at 24 h post-PDT, indicating that the induction of TRAIL receptors is time-consuming. However, TRAIL with short serum half-life was excluded from the blood within 0.5 h after injection, whereas the cytotoxicity of the same amount of IgBD-TRAIL persisted at least 24 h (Figure 1A). Although PDT increased the *in vitro* cytotoxicity of IgBD-TRAIL and TRAIL to similar degrees (Figure 4E, Figure S7B), the combination of long-acting IgBD-TRAIL and PDT induced greater apoptosis than did by TRAIL with short serum half-life combined with PDT in tumor grafts (Figure 5B, Figure 7B). On the other hand, in addition to sensitizing CRC cells, *in vivo* PDT usually damaged the vascular system of tumor grafts, which would induce super-enhanced permeability and retention effects, thus facilitating the entry of drugs into the tumor bed 32. In fact, PDT-induced hemorrhage accompanied by an increase in tumor uptake of TRAIL proteins was observed in both LS174T and HT29 tumor grafts (Figure 5A, Figure 7A). However, the tumor uptake of long-acting IgBD-TRAIL was much greater than that of TRAIL with short serum half-life. Consequently, combination of IgBD-TRAIL, but not TRAIL and PDT, efficiently eradicated tumor grafts of CRC cells with chemotherapeutic MDR and TRAIL resistance (Figure 6C, Figure 7C).

To further evaluate the contribution of PDT to overcoming chemotherapeutic MDR of CRC cells, we compared the expression levels of ABC transporters in LS174T cells treated with or without PDT. Of the ABC transporters, at least ABCB, ABCC, and ABCG subfamilies are involved in MDR of cancer cells 33. As shown in Figure S14 and Table S4, both up- and downregulations of MDR-associated ABC transporters were observed in LS174T cells treated with PDT. Upregulated ABC transporters included ABCB1 (p-glycoprotein, pGP), ABCB10, ABCC1-5 (multidrug resistance-associated protein 1-5, MRP1-5), and ABCC9, 10, and 11. Downregulated ABC transporters included ABCB4, ABCB6-9, ABCC6, and ABCG2 (breast cancer resistance protein 1, BCRP1). In the enterocytes, pGP, MRP1-5, and BCRP1 are in charge of efflux of chemical drugs 4. Except for BCRP1, Ze-IR700-mediated PDT increased all other six efflux transporters in LS174T cells (Figure S14, Table S4), which might increase the chemotherapeutic MDR of CRCs. These results suggested that the combination of chemical drugs and PDT might have little synergistic antitumor effect on CRC with MDR. As PDT induced apoptosis of CRC cells (Figure S5B), the combination of protein-based apoptosis inducers, including TRAIL, Fas ligand (Fas L), and tumor necrosis factor α (TNFα), theoretically has greater potential to overcome chemotherapeutic MDR of CRC. In fact, PDT significantly increased death receptor DR4 and DR5 for TRAIL, Fas for Fas L and TNFRSF1A for TNFα (Figure 4C and Table S3), providing the molecular basis for synergy between PDT and TRAIL, Fas L, and TNFα in the killing of CRC cells. However, owing to the inefficacy of soluble Fas L in apoptosis induction 34 and the systematic toxicity of TNFα 35, potent TRAIL without dose-limited toxicity must be the best choice for combination with PDT in cancer therapy.

Taken together, our results demonstrated that Ze-IR700-mediated PDT drastically sensitized LS174T and HT29 cells to TRAIL proteins by upregulating death receptors and that the combination of the long-acting IgBD-TRAIL and PDT efficiently eradicated tumor grafts of both CRC cells. As Ze-IR700-mediated PDT extensively sensitized CRC cells to TRAIL proteins (Figure S7C), along with the development of endoscope-assisted laser delivery technology, tumor cell-targeted PDT combined with long-acting TRAIL takes great potential for combating unresectable primary as well as metastatic CRC. Considering the good biosafety of TRAIL and PDT in patients, it is valuable to drive the clinical translation of this combination strategy in CRC precision therapy. Notably, although the combination therapy of IgBD-TRAIL and PDT induced transient body weight decrease in mice bearing CRC tumor grafts (Figure S13), the biosafety of this combination strategy should be further evaluated in future. In addition to TRAIL proteins, it is also interesting to combine PDT with other format of TRAIL, such as oncolytic virus-mediated TRAIL 36 and stem cell-delivered TRAIL 37, 38 for CRC-targeted therapy.

## Supplementary Material

Supplementary figures and tables.
