# Supplementary material for: Combination of long-acting TRAIL and tumor cell-targeted photodynamic therapy as a novel strategy to overcome chemotherapeutic multidrug resistance and TRAIL resistance of colorectal cancer
Source: Theranostics. 2021 Feb 25;11(9):4281–97. doi: 10.7150/thno.51193 (PMC7977453; doi:10.7150/thno.51193)
Supplement: Supplementary file 1 — Supplementary figures and tables. [file thnov11p4281s1.pdf]

Supplementary materials

Combination of long-acting TRAIL and tumor cell-targeted photodynamic therapy as a novel strategy to overcome chemotherapeutic multidrug resistance and TRAIL resistance of colorectal cancer

Tianshan She[1]*, Qiuxiao Shi[1]*, Zhao Li[1]*, Yanru Feng[1], Hao Yang[1,2], Ze Tao[1], Heng Li[1], Jie Chen[1], Shisheng Wang[3], Yan Liang[4], Jingqiu Cheng[1,2]#, Xiaofeng Lu[1,2]#

1 Key Lab of Transplant Engineering and Immunology, MOH, Regenerative Medical Research Center, West China Hospital, Sichuan University, Chengdu 610041, China
2 Frontiers Science Center for Disease-related Molecular Network, West China Hospital, Sichuan University, Chengdu 610041, China
3 West China-Washington Mitochondria and Metabolism Research Center, West China Hospital, Sichuan University, Chengdu 610041, China
4 Histopathology Platforms of Experimental Center, West China Hospital, Sichuan University, Chengdu 610041, China

* These authors contributed equally to this work.

# Correspondence should be addressed: xiaofenglu@scu.edu.cn (XF Lu) or jqcheng@scu.edu.cn (JQ Chen)

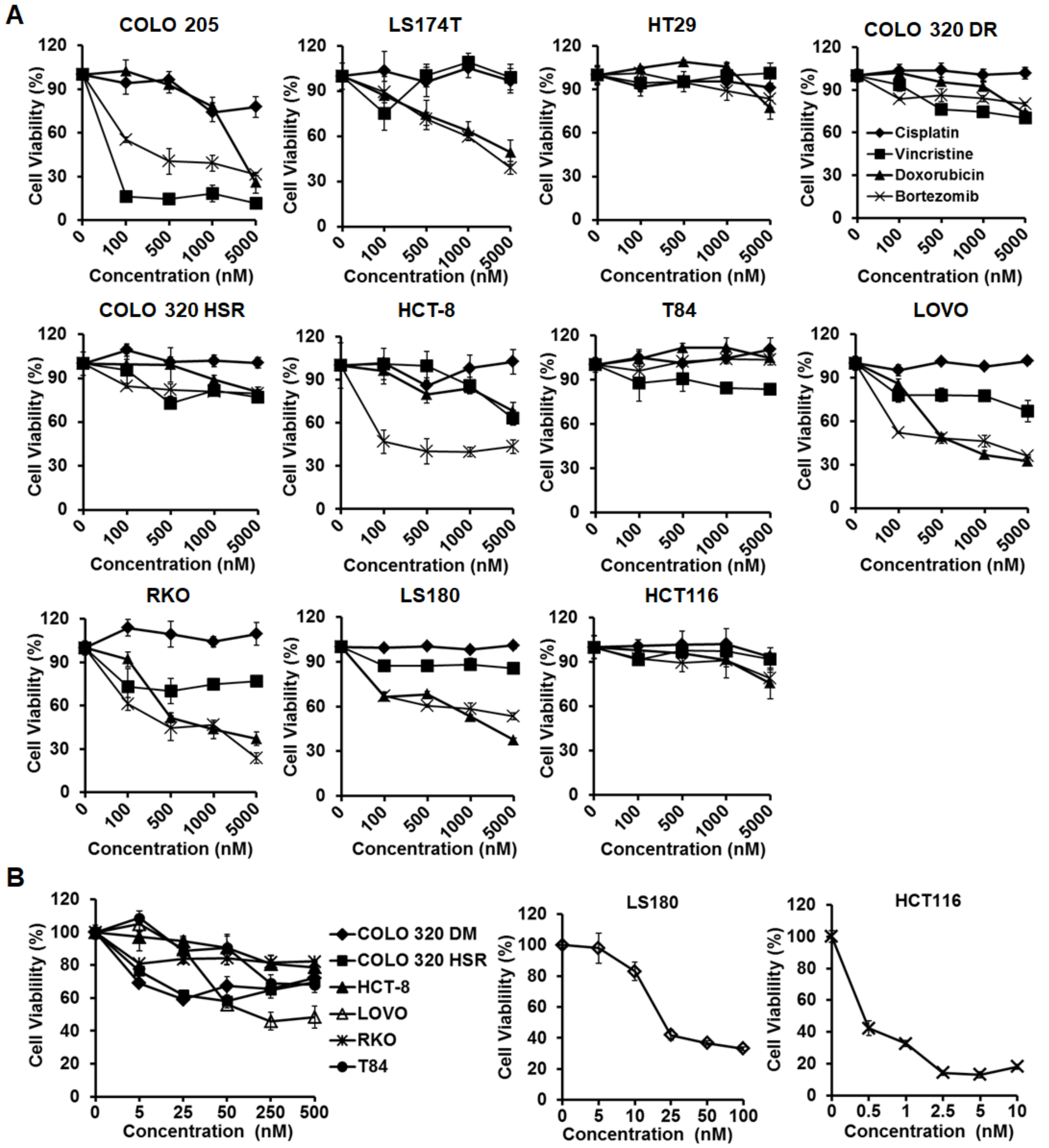

Figure S1 Cytotoxicity of chemical drugs (A) and TRAIL (B) in CRC cells. CRC cells were treated with chemicals (0-5000 nM) or TRAIL (0-500 nM) overnight followed by measuring the survival cells using CCK8. The viability of cells treated with PBS was considered 100%.

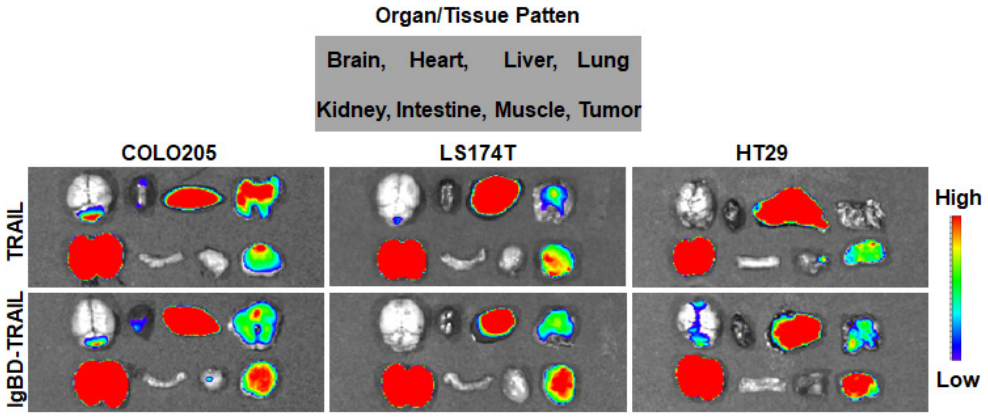

Figure S2 Tissue distribution of TRAIL proteins in mice bearing COLO 205, LS174T, and HT29 tumor grafts. CF750-labeled TRAIL or IgBD-TRAIL (5 mg/kg) was intravenously injected into the mice bearing tumor grafts. Some organs/tissues were collected and scanned using an IVIS optical imaging system at 4 h postinjection.

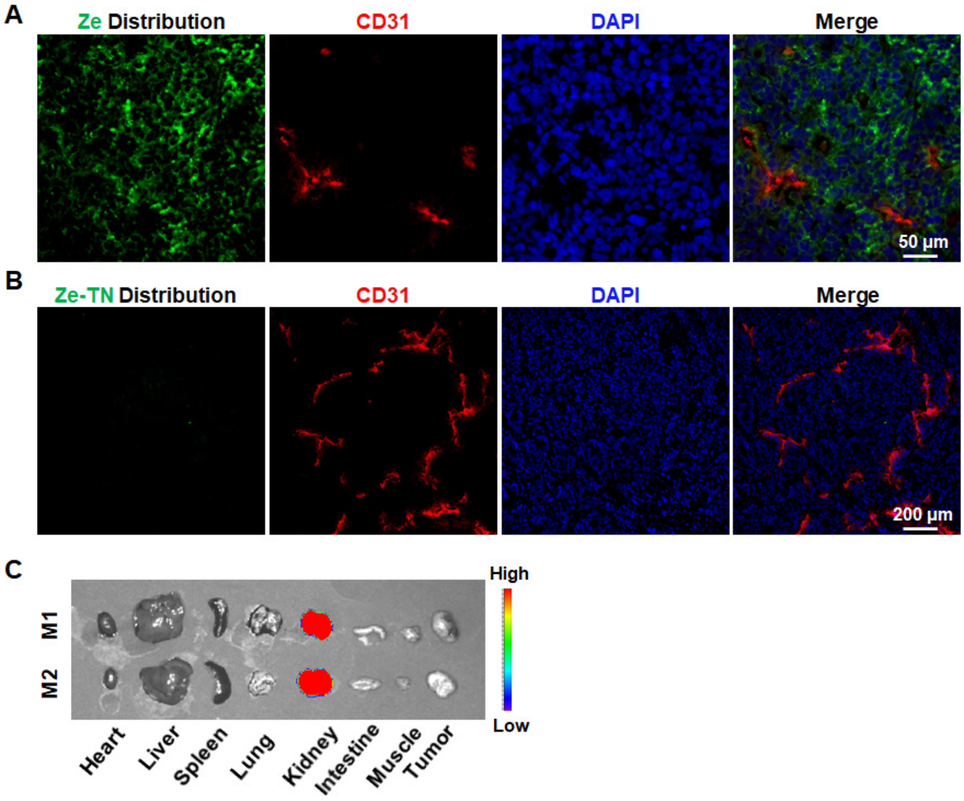

Figure S3 Cellular and tissue distribution of Ze affibody digested with (Ze-TN) or without (Ze) trypsin. **A, B.** Cellular distribution of Ze affibody digested with (B) or without (A) trypsin in LS174T tumor grafts. FAM-labeled affibody was intravenously injected into mice bearing LS174T tumor grafts. The tumor grafts were collected at 4 h postinjection followed by sectioning under frozen conditions. The tumor vessels were indicated by using antibody against CD31, and the nuclei were visualized by DAPI staining. **C.** Tissue distribution of Ze affibody digested with trypsin in mice bearing LS174T tumor grafts. CF750-labeled Ze affibody was digested with trypsin prior to injection into mice (N=2). Organs and tissues were collected at 4 h post injection and scanned using an IVIS optical imaging system.

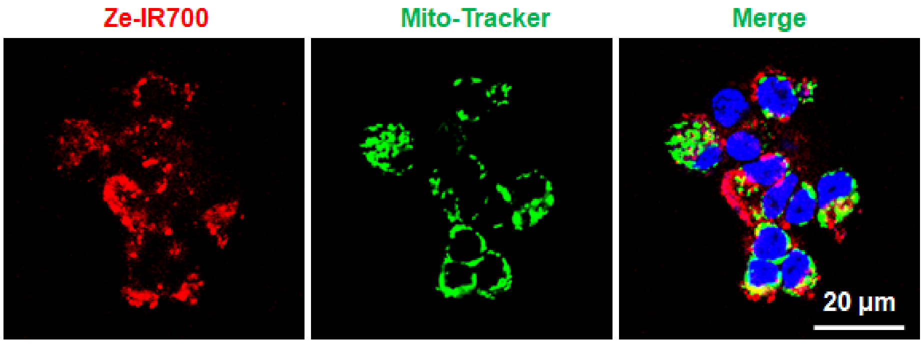

Figure S4 Localization of Ze-IR700 in the mitochondria of LS174T cells.

Ze-IR700 (red) and Mito-Tracker (green) were added into cells and incubated for 0.5 h followed by observation under a laser confocal microscope.

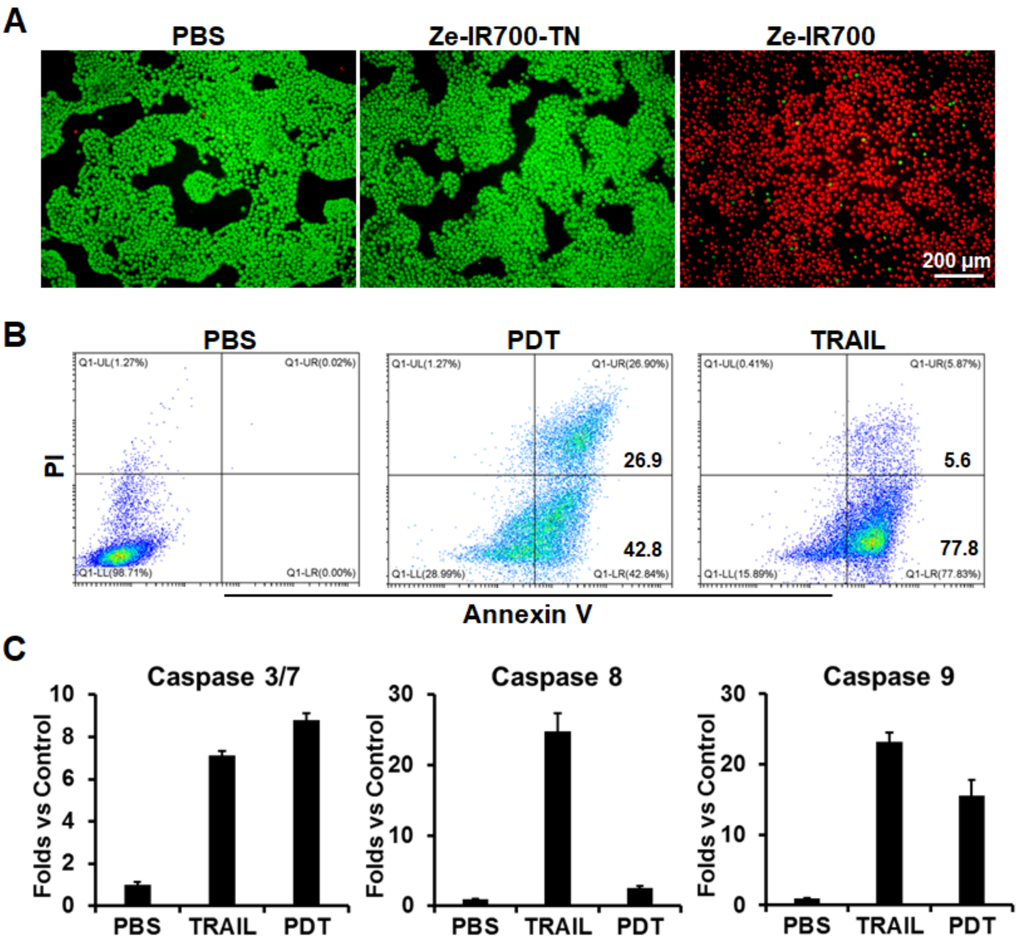

Figure S5 Death of CRC cells treated with Ze-IR700-mediated PDT or TRAIL proteins. A. PDT-mediated death of HT29 cells. Cells were treated with Ze-IR700- or trypsinized Ze-IR700 (Ze-IR700-TN)-mediated PDT followed by dual staining with SYTO 9 (indicating live cells) and PI (indicating dead cells). **B.** Flow cytometry analysis of apoptosis of LS174T cells. **C.** Involvement of caspases in the apoptosis of LS174T cells.

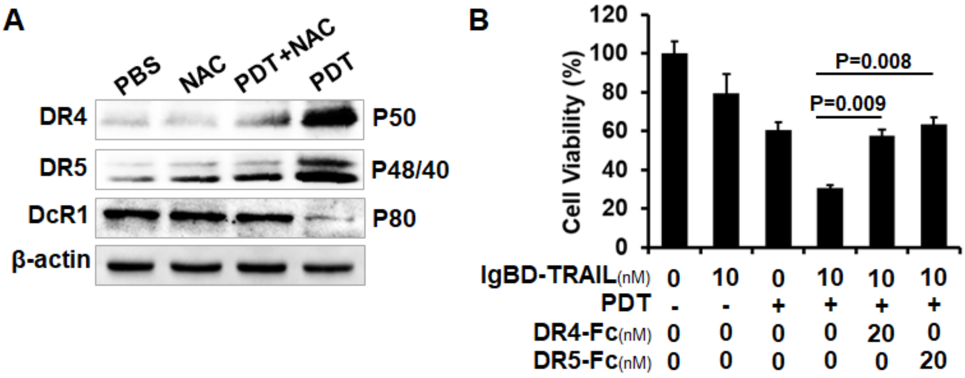

Figure S6 Regulation of death receptors and decoy receptor mediated by PDT(A) and inhibition of soluble death receptors on cell death induced by PDT (B). A. Western blot of death receptors (DR4 and DR5) and decoy receptor (DcR1) in cells treated with or without PDT in the presence or absence of antioxidant NAC. **B.** Reduction of PDT-induced death of LS174T cells by addition of soluble death receptors (DR4-Fc and DR5-Fc) in to the cells.

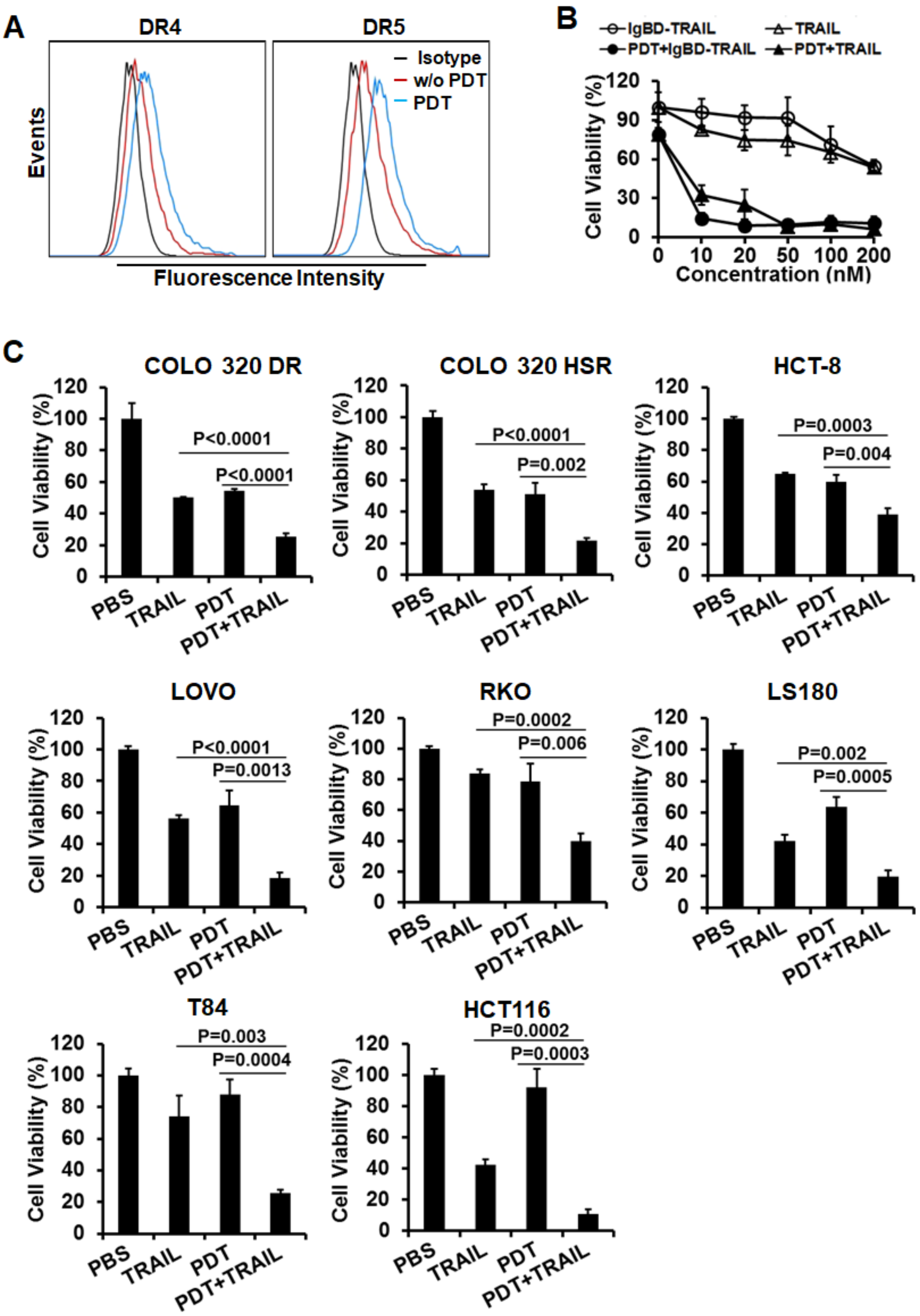

Figure S7 Sensitization of CRC cells to TRAIL proteins by PDT. A. Upregulation of DR4 and DR5 in HT29 cells by PDT. **B** and **C.** Synergistic killing of HT29 (B) and other CRC cells (C) between TRAIL proteins and PDT. CRC cells were pretreated with 0.5-2 μM Ze-IR700-mediated PDT followed by treatment with different concentrations (0-200 nM for HT29; 500 nM for COLO 320 DR, T84, HCT-8, and RKO cells; 250 nM for COLO 320 HSR cells; 50 nM for LS180 and LOVO cells, 0.5 nM for HCT116 cells) of TRAIL proteins overnight.

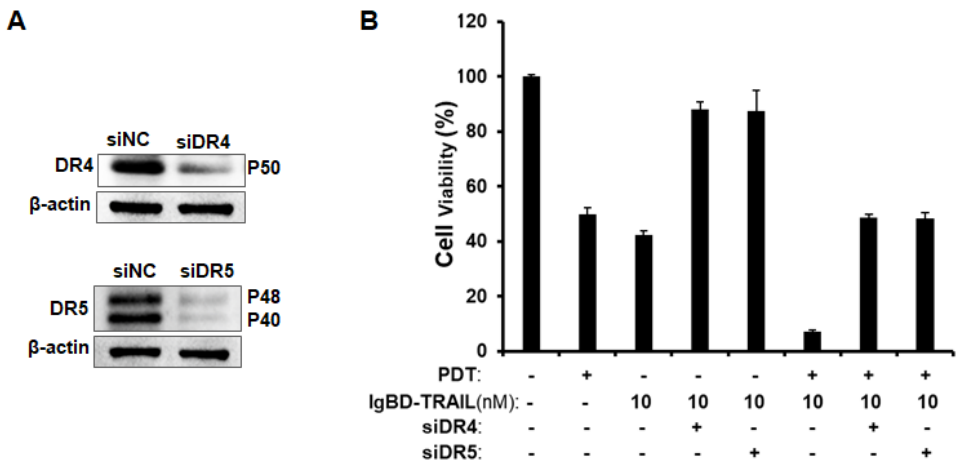

Figure S8 Abrogation of synergy between PDT and TRAIL in killing HCT116 cells by downregulation of death receptors. A. Western blot of DR4 and DR5 in HCT116 cells transfected with siRNAs specific for DR4 (siDR4) or DR5 (siDR5) or negative control siNC siRNA**. B.** Cytotoxicity of IgBD-TRAIL combined with or without PDT in HCT116 cells transfected with or without siRNAs. For western blot analysis, 6×10^4 cells were inoculated in 24-well plate followed by addition of 75nM siRNA into the cells. About 48 h later, cells were collected for western blot analysis and cytotoxicity assays.

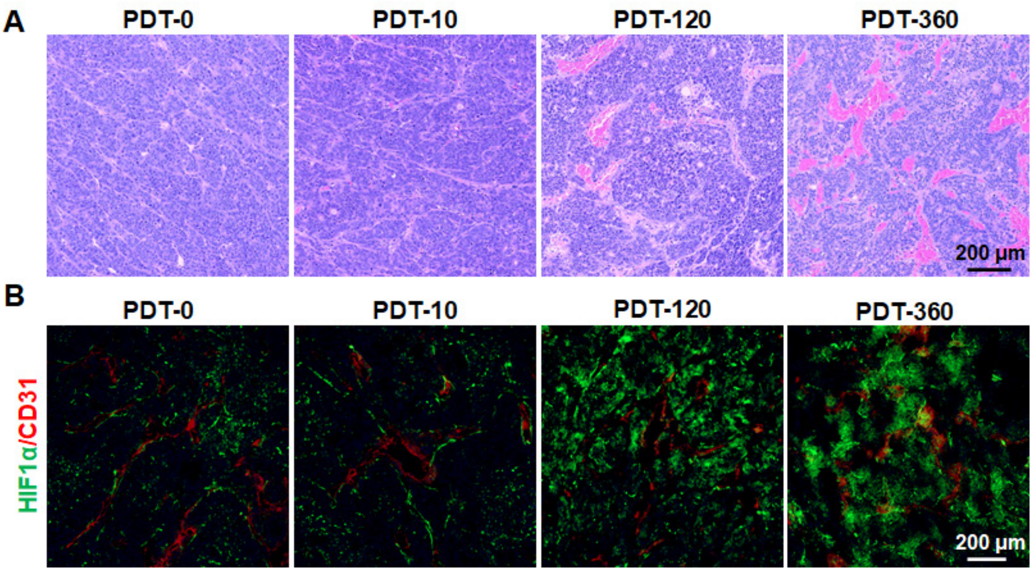

Figure S9 PDT-induced damage of tumor vessels (A) and hypoxia (B) in LS174T tumor grafts. Tumor tissues were collected and sectioned at 0-, 10-, 120-, or 360-min post-PDT followed by H&E staining (A) or immunofluorescence (B) of HIF1α and CD31. The nuclei were illustrated by DAPI staining.

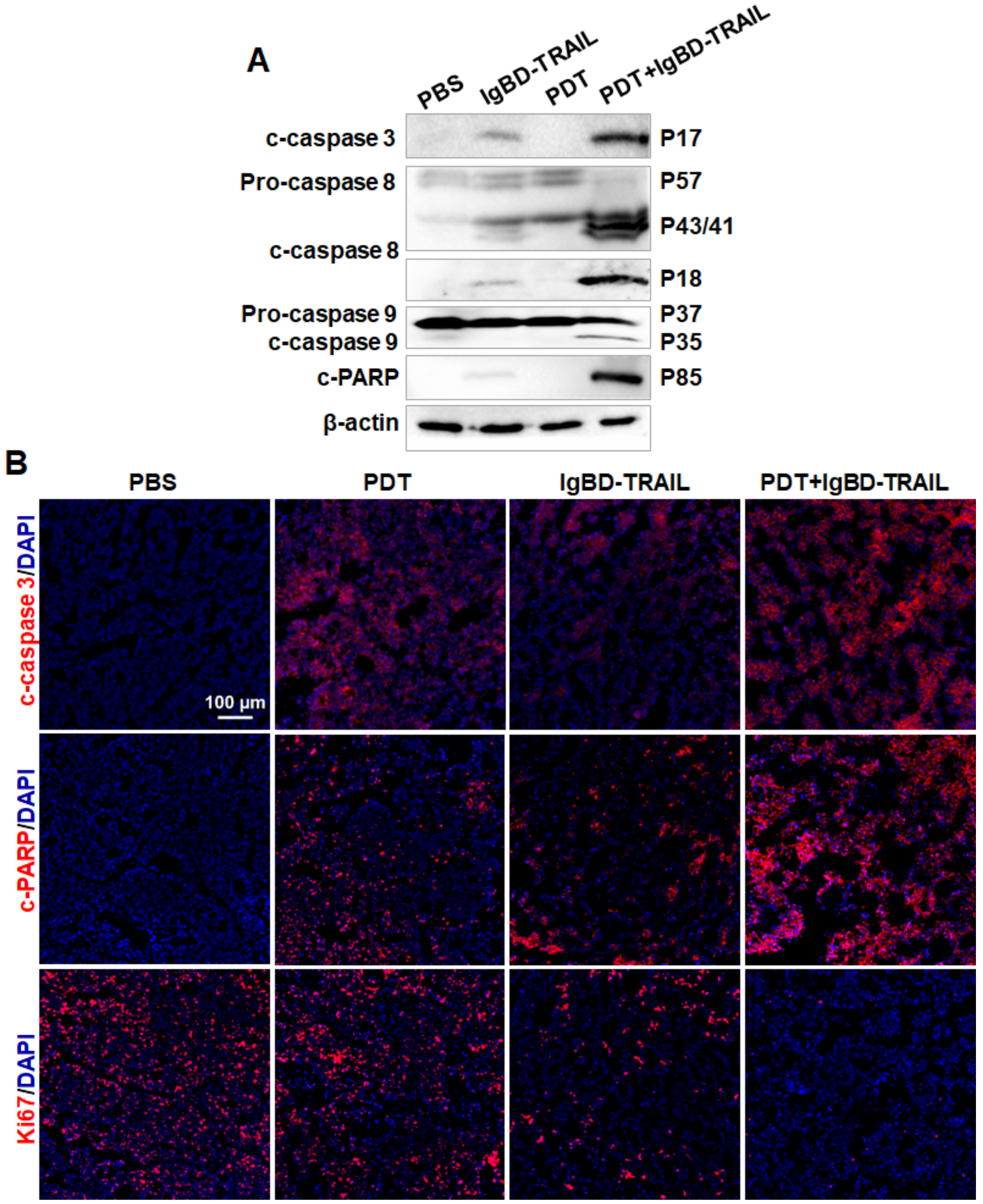

Figure S10 Involvement of caspase pathways in combating CRCs mediated by PDT and IgBD-TRAIL combination therapy. A. Western blot of cleaved caspase 3(c-caspase 3), cleaved canspase 8 (c-caspase 8), caspase 9 (pro-caspase 9, c-caspase 9) and cleaved PARP (c-PARP) in LS174T tumor cells. β-actin was used as control. **B.** Immunofluorescence of cleaved caspase 3 (c-caspase 3), cleaved PARP (c-PARP), and Ki67 in tumor grafts.

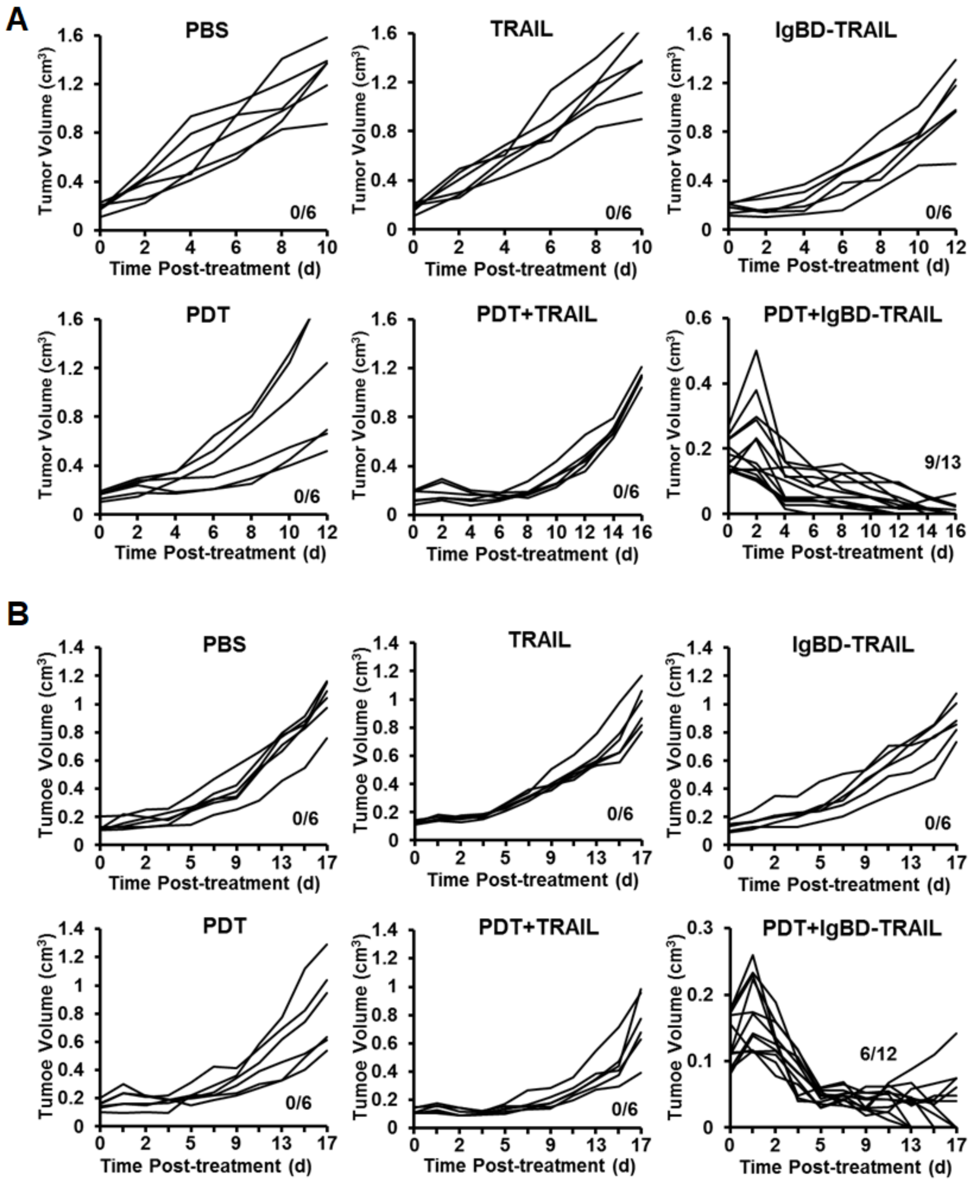

Figure S11 Perspective growth curves of LS174T (A) or HT29 (B) tumor grafts treated with monotherapy or combination therapy of TRAIL proteins and Ze-IR700-mediated PDT. The numbers of tumor-free mice at the end of observation are indicated.

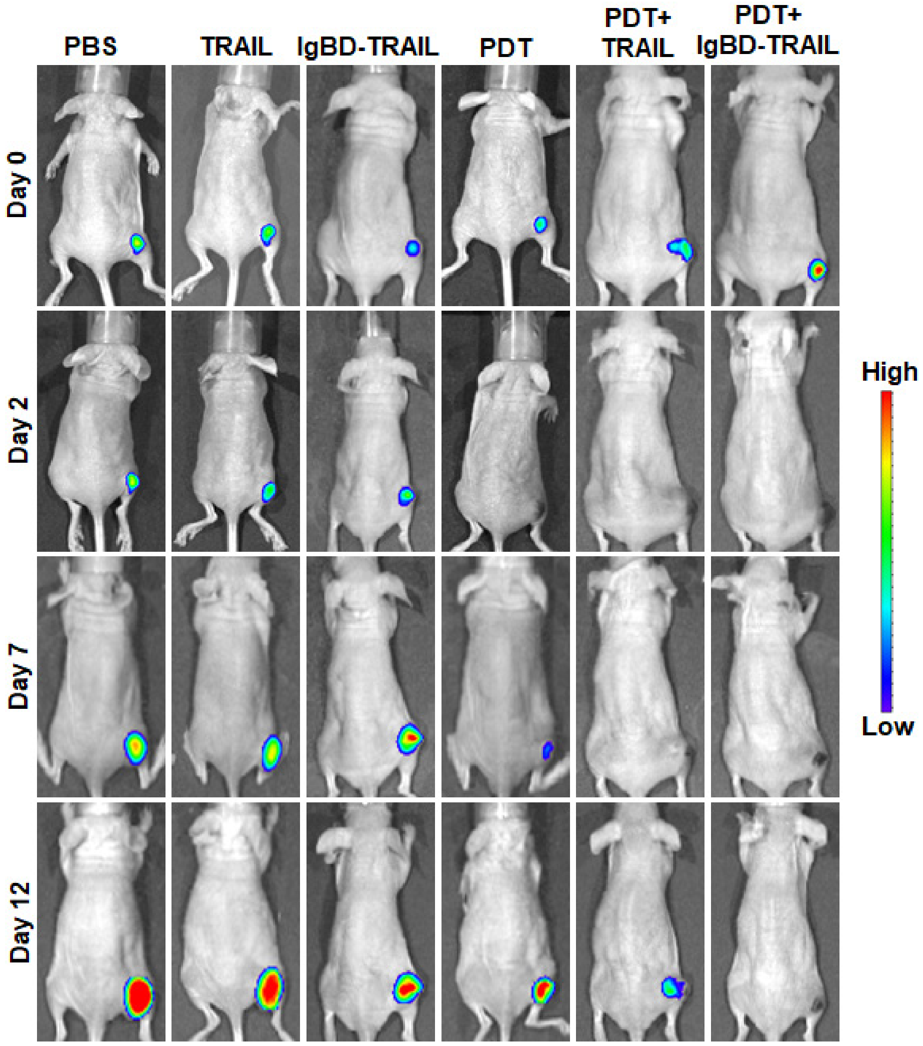

Figure S12 Dynamic optical imaging of mice bearing HT29 tumor grafts treated with monotherapy or combination therapy of TRAIL proteins and Ze-IR700-mediated PDT.

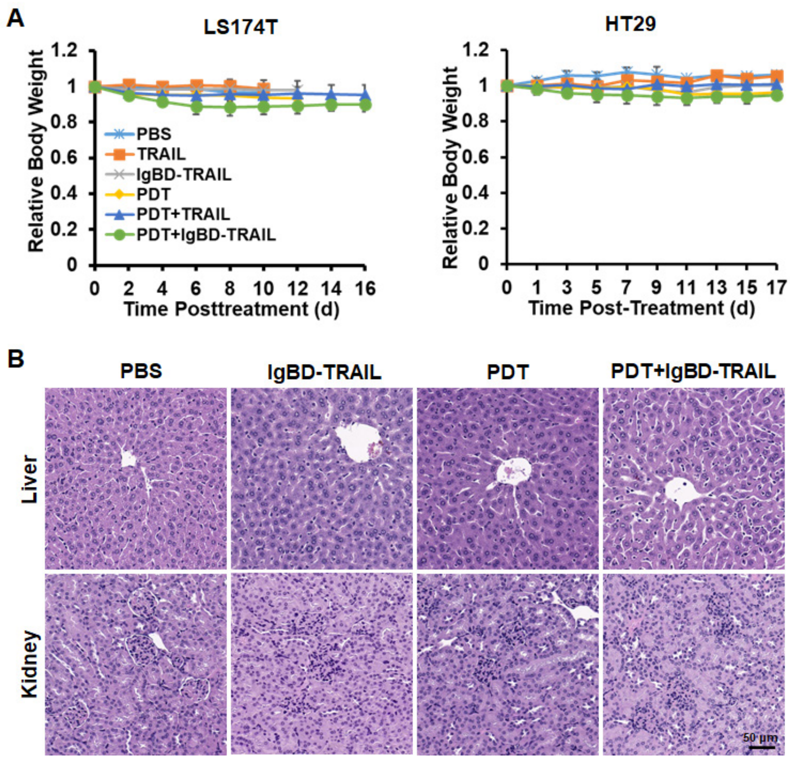

Figure S13 Variation of body weight of mice bearing LS174T or HT29 tumor grafts (A) and histochemistry of liver and kidney of mice bearing LS174T tumor grafts (B). The body weights of mice were recorded every day during treatment. The livers and kidneys of mice bearing LS174T tumor grafts were collected at the end of observation for histochemistry analysis.

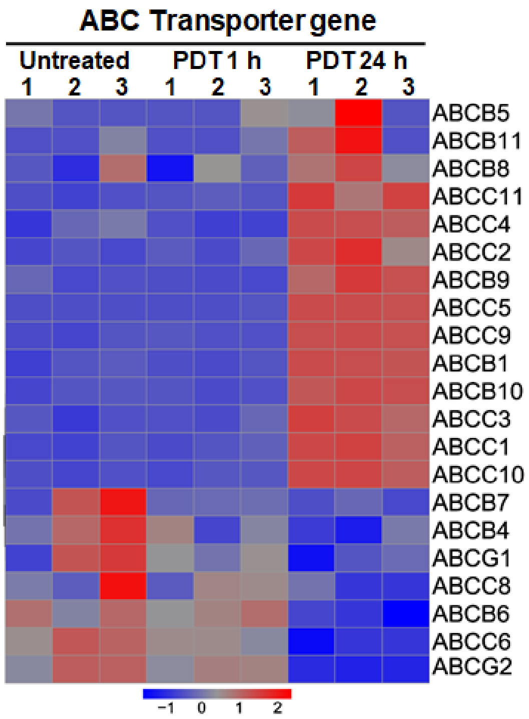

Figure S14 Expression profile of MDR-associated ABC transporter genes in LS174T cells treated with or without PDT.

Table S1 Fold change of pro-apoptotic genes in LS174T cells after treatment with PDT for 1 h or 24 h. Fold change is expressed as the ratio of the log 2 value of gene expression in PDT-treated cells to that of untreated cells. (**, *P* < 0.01; **, *P* < 0.05)

Gene Symbol	Gene Name	PDT 1h	Up/Down	PDT 24h	Up/Down	Function
**APAF1**	Apoptotic peptidase activating factor 1	0.19		0.70	↑ **	Caspase activator
**ATM**	ATM serine/threonine kinase	0.31	↑ *	1.44	↑ **	Positive regulation of apoptosis
**BAD**	BCL2 associated agonist of cell death	-0.25	↓ *	-0.62	↓ **	Pro-apoptotic/Positive regulation of apoptosis
**BAK1**	BCL2 antagonist/killer 1	-0.09		-0.76	↓ **	Pro-apoptotic/Positive regulation of apoptosis
**BAX**	BCL2 associated X, apoptosis regulator	-0.10		-0.25	↓ **	Pro-apoptotic/Caspase activator
**BCL2L11**	BCL2 like 11	0.33	↑ **	-0.14	↓ **	Pro-apoptotic
**BID**	BH3 interacting domain death agonist	-0.12		-0.87	↓ **	Pro-apoptotic/Positive regulation of apoptosis
**BOK**	BCL2 family apoptosis regulator BOK	0.04		-0.45	↓ **	Pro-apoptotic
**CASP10**	Caspase 10	-0.12		0.13	↑ *	Pro-apoptotic/Positive regulation of apoptosis
**CASP2**	Caspase 2	-0.03		0.70	↑ **	Pro-apoptotic/Positive regulation of apoptosis
**CASP8**	Caspase 8	-0.12		1.04	↑ **	Pro-apoptotic/Positive regulation of apoptosis
**CASP9**	Caspase 9	-0.05		0.03	↑ *	Pro-apoptotic/Positive regulation of apoptosis
**CHUK**	Conserved helix-loop-helix ubiquitous kinase	-0.06		0.55	↑ **	Pro-apoptotic/Positive regulation of apoptosis
**CTSB**	Cathepsin B	0.09		-0.79	↓ **	Pro-apoptotic/Positive regulation of apoptosis
**CTSC**	Cathepsin C	0.03		0.50	↑ **	Pro-apoptotic/Positive regulation of apoptosis
**CTSD**	Cathepsin D	0.09		0.54	↑ **	Pro-apoptotic/Positive regulation of apoptosis
**CTSF**	Cathepsin F	0.12		-0.70	↓ **	Pro-apoptotic/Positive regulation of apoptosis
**CTSH**	Cathepsin H	-0.11		-0.80	↓ **	Pro-apoptotic/Positive regulation of apoptosis
**CTSK**	Cathepsin K	0.11		-0.80	↓ **	Pro-apoptotic/Positive regulation of apoptosis
**CTSS**	Cathepsin S	-0.23		-0.53	↓ **	Pro-apoptotic/Positive regulation of apoptosis
**CTSV**	Cathepsin V	0.01		-0.50	↓ **	Pro-apoptotic/Positive regulation of apoptosis
**CTSZ**	Cathepsin Z	0.02		-0.83	↓ **	Pro-apoptotic/Positive regulation of apoptosis
**CYCS**	Cytochrome c	-0.14		-0.32	↓ **	Pro-Apoptotic
**DDIT3**	DNA damage inducible transcript 3	0.34	↑ **	1.05	↑ **	Pro-apoptotic/Positive regulation of apoptosis
**DFFA**	DNA fragmentation factor subunit alpha	0.02		0.38	↑ **	Pro-apoptotic/Positive regulation of apoptosis

Table S1 continued

Gene Symbol	Gene Name	PDT 1 h	Up/Down	PDT 24 h	Up/Down	Function
**DIABLO**	Diablo IAP-binding mitochondrial protein	-0.09		-0.23	↓ **	Pro-apoptotic
**DNM1L**	Dynamin 1 like	0.04		0.43	↑ *	Pro-apoptotic/positive regulation of apoptosis
**ENDOG**	Endonuclease G	-0.13		-0.35	↓ **	Pro-apoptotic/positive regulation of apoptosis
**ERN1**	Endoplasmic reticulum to nucleus signaling 1	0.26	↑ *	1.67	↑ **	Pro-apoptotic/positive regulation of apoptosis
**GADD45A**	Growth arrest and DNA damage inducible alpha	0.02		-1.26	↓ **	Positive regulation of apoptosis
**GADD45B**	Growth arrest and DNA damage inducible beta	0.32	↑ **	-3.44	↓ **	Positive regulation of apoptosis
**GADD45G**	Growth arrest and DNA damage inducible gamma	0.49	↑ *	-0.89	↓ **	Positive regulation of apoptosis
**HTRA2**	HtrA serine peptidase 2	0.00		-0.88	↓ **	Pro-apoptotic/positive regulation of apoptosis
**LATS1**	Large tumor suppressor kinase 1	0.17		1.25	↑ **	Pro-apoptotic/positive regulation of apoptosis
**LATS2**	Large tumor suppressor kinase 2	0.26	↑ *	-0.16	↓ **	Pro-apoptotic/positive regulation of apoptosis
**MFN2**	Mitofusin 2	0.07		0.63	↑ **	Pro-apoptotic/positive regulation of apoptosis
**PIDD1**	P53-induced death domain protein 1	0.12		-0.37	↓ **	Pro-apoptotic/positive regulation of apoptosis
**PMAIP1**	Phorbol-12-myristate-13-acetate-induced protein 1	0.36	↑ **	-0.13	↓ **	Pro-apoptotic/positive regulation of apoptosis
**SAV1**	Salvador family WW domain containing protein 1	0.03		0.52	↑ **	Positive regulation of apoptotic process
**TP53**	Tumor protein p53	0.11		1.92	↑ **	Pro-apoptotic/Positive regulation of apoptosis
**TP53AIP1**	TP53 regulated apoptosis inducing protein 1	0.87	↑ **	-0.89	↓ **	Pro-apoptotic/Positive regulation of apoptosis
**TRADD**	TNFRSF1A associated via death domain	-0.01		-0.25	↓ *	Pro-apoptotic/Positive regulation of apoptosis

Table S2 Fold change of anti-apoptotic genes in LS174T cells after treatment with PDT for 1 h or 24 h. Fold change is expressed as the ratio of the log 2 value of gene expression in PDT-treated cells to that of untreated cells. (**, *P* < 0.01; **, *P* < 0.05)

Gene Symbol	Gene Name	PDT 1 h	Up/Down	PDT 24 h	Up/Down	Function
**AKT1**	AKT serine/threonine kinase 1	0.01		-0.04	↓ **	Anti-Apoptotic/Positive regulation of apoptosis
**ATF4**	Activating transcription factor 4	0.08		0.62	↑ **	Negative regulation of apoptosis
**BCL2**	BCL2 apoptosis regulator	0.42		1.64	↑ **	Anti-apoptotic/Negative regulation of apoptosis
**BCL2L1**	BCL2 like 1	0.01		0.08	↑ *	Anti-apoptotic
**BECN1**	Beclin 1	0.03		0.43	↑ *	Anti-apoptotic
**BIRC2**	Baculoviral IAP repeat containing 2	0.10		0.60	↑ **	Anti-apoptotic
**BIRC5**	Baculoviral IAP repeat containing 5	0.03		-0.23	↓ **	Anti-apoptotic
**BIRC6**	Baculoviral IAP repeat containing 6	0.20		0.97	↑ **	Anti-apoptotic
**BRAF**	B-RAF proto-oncogene	0.07		0.98	↑ **	Negative regulation of apoptosis
**CFLAR**	CASP8 and FADD like apoptosis regulator	0.05		0.77	↑ **	Anti-apoptotic/Negative regulation of apoptosis
**ELF1**	E74 like ETS transcription factor 1	0.09		1.11	↑ **	Positive regulation of transcription
**ELF2**	E74 like ETS transcription factor 2	0.02		0.60	↑ **	Positive regulation of transcription
**ELF4**	E74 like ETS transcription factor 4	-0.01		0.44	↑ **	Positive regulation of transcription
**FAIM**	Fas apoptotic inhibitory molecule	-0.10		2.44	↑ **	Negative regulation of apoptotic process
**HRAS**	HRAS proto-oncogene, GTPase	-0.05		1.06	↑ **	Negative regulation of apoptotic process
**IKBKG**	I-kappa-B kinase subunit gamma	0.07		0.62	↑ **	Anti-apoptotic/Negative regulation of apoptosis
**IL3RA**	Interleukin 3 receptor subunit alpha	-0.17		-2.57	↓ **	Anti-apoptotic/Negative regulation of apoptosis
**ITPR1**	Inositol 1,4,5-trisphosphate receptor type 1	0.20		-0.26	↓ **	Anti-apoptotic/Negative regulation of apoptosis
**ITPR3**	Inositol 1,4,5-trisphosphate receptor type 3	0.16		0.44	↑ *	Anti-apoptotic/Negative regulation of apoptosis
**MAP2K1**	Mitogen-activated protein kinase kinase 1	0.01		-0.41	↓ **	Anti-apoptotic/Negative regulation of apoptosis
**MAP2K2**	Mitogen-activated protein kinase kinase 2	-0.08		-0.96	↓ **	Anti-apoptotic/Negative regulation of apoptosis
**MAP2K7**	Mitogen-activated protein kinase kinase 7	0.04		-0.08	↓ **	Anti-apoptotic/Negative regulation of apoptosis
**MAPK1**	Mitogen-activated protein kinase 1	0.00		0.60	↑ **	Anti-apoptotic/Negative regulation of apoptosis
**MAPK3**	Mitogen-activated protein kinase 3	-0.08		-2.03	↓ **	Anti-apoptotic/Negative regulation of apoptosis

Table S2 Continued

Gene Symbol	Gene Name	PDT 1 h	Up/Down	PDT 24 h	Up/Down	Function
**MAPK8**	Mitogen-activated protein kinase 8	0.02		0.75	↑ **	Anti-apoptotic/Negative regulation of apoptosis
**MAPK9**	Mitogen-activated protein kinase 9	-0.02		0.08	↑ **	Anti-apoptotic/Negative regulation of apoptosis
**MCL1**	MCL1 apoptosis regulator	0.28	↑ **	-0.33	↓ **	Negative regulation of apoptosis
**NFKB1**	Nuclear factor kappa B subunit 1	0.03		1.28	↑ **	Anti-apoptotic/Negative regulation of apoptosis
**NRAS**	NRAS proto-oncogene	-0.06		0.63	↑ **	Anti-apoptotic/Negative regulation of apoptosis
**PARP2**	Poly(ADP-ribose) polymerase 2	-0.13		0.08	↑ *	DNA repair/Positive regulation of transcription
**PARP3**	Poly(ADP-ribose) polymerase 3	-0.04		0.72	↑ **	DNA repair/Positive regulation of transcription
**PARP4**	Poly(ADP-ribose) polymerase 4	0.02		1.08	↑ **	DNA repair/Positive regulation of transcription
**PDPK1**	3-phosphoinositide dependent protein kinase 1	0.13		1.58	↑ **	Anti-apoptotic/Negative regulation of apoptosis
**PIK3CA**	Phosphoinositide-3-kinase subunit alpha	0.12		0.69	↑ **	Anti-apoptotic/Negative regulation of apoptosis
**PIK3CB**	Phosphoinositide-3-kinase subunit beta	0.08		0.03	↑ *	Anti-apoptotic/Negative regulation of apoptosis
**PIK3CD**	Phosphoinositide-3-kinase subunit delta	0.14		-1.93	↓ *	Anti-apoptotic/Negative regulation of apoptosis
**PIK3R1**	Phosphoinositide-3-kinase regulatory subunit 1	0.07		1.19	↑ **	Anti-apoptotic/Negative regulation of apoptosis
**PIK3R2**	Phosphoinositide-3-kinase regulatory subunit 2	0.03		-0.42	↓ **	Anti-apoptotic/Negative regulation of apoptosis
**PIK3R3**	Phosphoinositide-3-kinase regulatory subunit 3	0.18		0.54	↑ **	Anti-apoptotic/Negative regulation of apoptosis
**PRDX2**	Peroxiredoxin 2	-0.02		-0.40	↓ **	Negative regulation of apoptosis
**PRDX4**	Peroxiredoxin 4	-0.17		-0.11	↓ **	Negative regulation of apoptosis
**RAF1**	Raf-1 proto-oncogene, serine/threonine kinase	0.11		0.60	↑ **	Positive regulation of cell proliferation
**RELA**	RELA proto-oncogene	-0.02		-0.16	↓ **	Negative regulation of apoptosis
**RIPK1**	Receptor interacting serine/threonine kinase 1	0.29	↑ **	0.68	↑ **	Pro-apoptotic/Positive regulation of apoptosis
**RNF157**	Ring finger protein 157	0.17		-0.23	↓ **	Negative regulation of apoptotisis
**TRAF1**	TNF receptor associated factor 1	0.06		1.12	↑ **	Anti-apoptotic/Negative regulation of apoptosis
**TUBA1B**	Tubulin alpha 1b	0.04		0.45	↑ **	Microtubule cytoskeleton organization
**XIAP**	X-linked inhibitor of apoptosis	0.05		1.43	↑ **	Anti-apoptotic/Negative regulation of apoptosis
**PTPN13**	Protein tyrosine phosphatase non-receptor type 13	0.19		0.52	↑ **	Negative regulation of apoptosis

Table S3 Fold change of death receptors and decoy receptors in LS174T cells after treatment with PDT for 1 h or 24 h. Fold change is expressed as the ratio of the log 2 value of gene expression in PDT-treated cells to that of untreated cells. (**, *P* < 0.01; **, *P* < 0.05)

Gene Symbol	Gene Name	PDT 1 h	Up/Down	PDT 24 h	Up/Down	Function
**TNFRSF10A**	TNF receptor superfamily member 10A	0.15	↑	0.66	↑ **	Pro-apoptotic/Death domain receptor
**TNFRSF10B**	TNF receptor superfamily member 10B	0.33	↑ **	0.83	↑ **	Pro-apoptotic/Death domain receptor
**TNFRSF10C**	TNF receptor superfamily member 10C	-0.04		-0.36	↓ **	Anti-apoptotic/Decoy receptor
**TNFRSF10D**	TNF receptor superfamily member 10D	0.26	↑ **	-1.05	↓ **	Anti-apoptotic/Decoy receptor
**TNFRSF1A**	TNF receptor superfamily member 1A	0.06		0.41	↑ *	Pro-apoptotic/Death domain receptor
**FAS**	TNF receptor superfamily member 6	-0.04		0.42		Pro-apoptotic//Death domain receptor
**TNFRSF6B**	TNF receptor superfamily member 6B	0.01		-0.93	↓ **	Anti-apoptotic/Decoy receptor
**TNFRSF21**	TNF receptor superfamily member 21	0.08		-0.29	↓ **	Pro-apoptotic//Death domain receptor
**TNFRSF25**	TNF receptor superfamily member 25	0.29		-1.80	↓ **	Pro-apoptotic/Death domain receptor
**EDAR**	TNF receptor superfamily member EDAR	0.19		-0.97	↓ **	Pro-apoptotic /Death domain receptor
**NGFR**	TNF receptor superfamily member 16	-0.10		-1.72	↓ **	Pro-apoptotic/Death domain receptor

Table S4 Fold change of MDR-associated ABC transporters in LS174T cells after treatment with PDT for 1 h or 24 h. Fold change is expressed as the ratio of the log 2 value of gene expression in PDT-treated cells to that of untreated cells. (**, $P < 0.01$; **, $P < 0.05$)

Gene Symbol	Gene Name	PDT 1 h	Up/Down	PDT 24 h	Up/Down	Function
**ABCB1**	P glycoprotein (pGP)	0.17		1.00	↑ **	Drug efflux transporter
**ABCB4**	ATP-binding cassette subfamily B member 4	-0.25		-1.58	↓ **	Phospholipids transporter
**ABCB5**	ATP-binding cassette subfamily B member 5	1.00		2.17	↑	Drug efflux transporter
**ABCB6**	ATP-binding cassette subfamily B member 6	0.15		-1.02	↓ **	Heme transporter
**ABCB7**	ATP-binding cassette subfamily B member 7	-0.11		-0.97	↓ **	Heme transporter
**ABCB8**	ATP-binding cassette subfamily B member 8	0.12		-0.46	↓ **	ATP hydrolysis transporter
**ABCB9**	ATP-binding cassette subfamily B member 9	0.11		-0.22	↓ **	Peptide transporter
**ABCB10**	ATP-binding cassette subfamily B member 10	0.19		1.34	↑ **	Heme transporter
**ABCB11**	ATP binding cassette subfamily B member 11	0.00		2.32	↑	Canalicular bile salt efflux transporter
**ABCC1**	Multidrug resistance-associated protein 1 (MRP1)	0.26	↑ *	0.34		Drug efflux transporter
**ABCC2**	Multidrug resistance-associated protein 2 (MRP2)	0.54		1.37	↑ **	Drug efflux transporter
**ABCC3**	Multidrug resistance-associated protein 3 (MRP3)	0.23		0.11		Drug efflux transporter
**ABCC4**	Multidrug resistance-associated protein 4 (MRP4)	-0.16		0.63		Drug efflux transporter
**ABCC5**	Multidrug resistance-associated protein 5 (MRP5)	0.22		1.60	↑ **	Drug efflux transporter
**ABCC6**	Multidrug resistance-associated protein 6 (MRP6)	-0.07		-1.96	↓ **	Drug efflux transporter
**ABCC8**	ATP-binding cassette subfamily C member 8	-0.19		-3.00	↓	ATP-sensitive K^+ channel regulator
**ABCC9**	ATP-binding cassette subfamily C member 9	0.31	↑ *	1.03	↑ **	ATP-sensitive K^+ channel regulator
**ABCC10**	Multidrug resistance-associated protein 7 (MRP7)	0.27		0.79	↑ **	Lipophilic anion transporter
**ABCC11**	Multidrug resistance-associated protein 8 (MRP8)	1.32		3.36	↑ **	Lipophilic anion transporter
**ABCG1**	ATP-binding cassette subfamily G member 1	0.08		-0.91	↓ **	Phospholipids transporter
**ABCG2**	Breast cancer resistant protein 1 (BCRP1)	-0.02		-3.30	↓ **	Drug efflux transporter
